# Sumoylation of RORγt regulates T_H_17 differentiation and thymocyte development

**DOI:** 10.1038/s41467-018-07203-z

**Published:** 2018-11-19

**Authors:** Zhiheng He, Jing Zhang, Zhaofeng Huang, Qian Du, Ning Li, Qiang Zhang, Yuan Chen, Zuoming Sun

**Affiliations:** 1 Division of Molecular Immunology, Beckman Research Institute of City of Hope, Duarte, 91010 CA USA; 2 Irell & Manella Graduate School of Biological Sciences, City of Hope, Duarte, 91010 CA USA; 3 Zhongshan School of Medicine, Sun Yat-sen University, Guangzhou, 510080 Guangdong China; 4 Department of Infectious Diseases, Huashan Hospital, Fudan Univerity, Shanghai, 200040 China; 5 Tianjin Medical University General Hospital, Tianjin Geriatrics Institute, Tianjin, 300052 China; 6 Division of Molecular Medicine, Beckman Research Institute of City of Hope, Duarte, 91010 CA USA

## Abstract

RORγt controls the differentiation of T_H_17 cells, which are mediators of autoimmune conditions such as experimental autoimmune encephalomyelitis (EAE). RORγt also regulates thymocyte development and lymph node genesis. Here we show that the function of RORγt is regulated by its sumoylation. Loss of *Sumo3*, but not *Sumo1*, dampens T_H_17 differentiation and delays the progression of thymic CD8^+^ immature single-positive cells (ISPs). RORγt is SUMO3-modified by E3 ligase PIAS4 at lysine 31 (K31), and the mutation of K31 to arginine in mice prevents RORγt sumoylation, leading to impaired T_H_17 differentiation, resistance to T_H_17-mediated EAE, accumulation of thymic ISPs, and a lack of Peyer’s patches. Mechanistically, sumoylation of RORγt-K31 recruits histone acetyltransferase KAT2A, which stabilizes the binding of SRC1 to enhance RORγt transcription factor activity. This study thus demonstrates that sumoylation is a critical mechanism for regulating RORγt function, and reveals new drug targets for preventing T_H_17-mediated autoimmunity.

## Abstract (web summary)

The transcription factor RORγt is essential for the differentiation of T_H_17 cells, thymocyte development and lymphoid organogenesis. Here the authors show that the function of RORγt is regulated by PIAS4-mediated sumoylation that stabilize the interaction with SRC1 and KAT2A, to enhance the transcriptional activity of RORγt.

## Introduction

The transcription factor RORγt directs the differentiation of T_H_17 cells, which secrete IL-17 and participate in both protective and pathological immunity^1^. The clearance of pathogens such as *Citrobacter rodentium* and fungus depends on robust protective T_H_17 immunity^2–6^. On the other hand, T_H_17 cells also mediate the pathological immune responses involved in autoimmune conditions, such as multiple sclerosis, colitis, and even autism, and the prevention of these conditions depends on inhibiting the formation and function of T_H_17 cells^7–12^. The critical function of RORγt has been demonstrated by severe immune deficiency in both mice^13^ and humans^14^ carrying mutated versions of the RORγt-encoding gene *Rorc*. In addition, RORγt enhances thymocyte survival and is thus essential for thymic T cell development. RORγt is also required for the biogenesis of secondary lymph tissues, including gut-associated Peyer’s patches^15–18^. Because RORγt is required for the generation of pathogenic T_H_17 cells responsible for autoimmunity, it is an attractive target for the development of drugs to control T_H_17-mediated immunological disorders^19,20^. It is thus important to understand the mechanisms regulating RORγt function.

As a member of the steroid nuclear receptor superfamily, RORγt has two conserved domains^21,22^: an amino-terminal DNA-binding domain and a carboxyl-terminal ligand-binding domain. The very carboxyl terminal of the ligand-binding domain is an activation function 2 (AF2) motif responsible for recruiting steroid receptor coactivator 1 (SRC1) to nuclear receptors, which is required for RORγt-mediated transactivation of genes essential for T_H_17 differentiation^23–25^. Because RORγt is a transcription factor, previous studies have focused on the transcriptional aspects of RORγt function. However, the post-translational mechanisms that regulate RORγt function have long been neglected.

Sumoylation is a type of post-translational modification in which small ubiquitin-related modifier (SUMO) proteins are covalently attached to the lysines of target proteins. Mammals usually express three SUMO proteins: SUMO1, SUMO2, and SUMO3, which share approximately 50% amino acid sequence identity. Sumoylation is a multi-step reaction that is sequentially catalyzed by a SUMO-activating E1 enzyme, the single conjugating E2 enzyme Ubc9, and an E3 ligase. Sumoylation controls many aspects of cellular function^26,27^ by regulating protein stability and by enabling new protein–protein interactions through the addition of new docking sites. Knockout of the E2 enzyme *Ubc9* affects thymic T cell development and the expansion of regulatory T cells^28,29^, implicating sumoylation as an important regulator of these two processes. However, the roles of sumoylation in other aspects of T cell development and function, including T_H_17 differentiation, remain unknown.

Here, we demonstrate that the loss of *Sumo3*, but not *Sumo1*, impairs T_H_17 differentiation and delays progression of thymic immature single-positive (ISP) CD8^+^ cells, which are similar to phenotypes observed in *Rorγt*^*−/*−^ mice. This work leads us to identify lysine 31 (K31) as a functional sumoylation site in RORγt. We find that mice expressing K31-mutant RORγt^K31R^ are incapable of being sumoylated at K31 and exhibit multiple defective RORγt-dependent functions, including differentiation of T_H_17, induction of T_H_17-dependent experimental autoimmune encephalomyelitis (EAE), the progression of thymic ISP, and development of Peyer’s patches. Additional data attribute these effects to the defective recruitment of histone acetyltransferase KAT2A, which impairs the interactions between RORγt and co-activator SRC1. Finally, we identify the E3 ligase responsible for RORγt sumoylation to be PIAS (protein inhibitor of activated STAT) proteins form the largest family of sumoylation E3 ligases^30^, as the E3 ligase PIAS4 is able to bind and sumoylate RORγt at K31, and knockdown of PIAS4 impairs RORγt-dependent T_H_17 differentiation and progression of ISP, phenocopying the effects observed in RORγt^K31R/K31R^ mice. Our study thus reveals sumoylation as a novel post-translational mechanism for regulating RORγt-dependent functions.

## Results

### *Sumo3*, but not *Sumo1*, stimulates T_H_17 differentiation

**Fig. 1:**
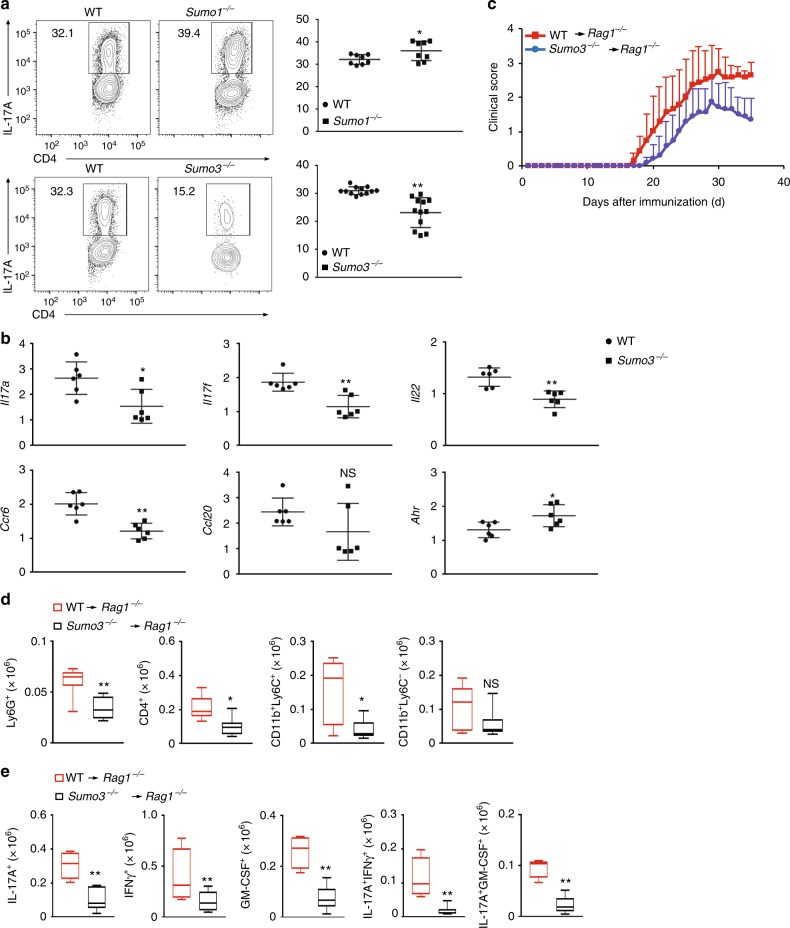
SUMO3, but not SUMO1, stimulates T_H_17 differentiation. **a** Representative flow cytometric analysis of intracellular IL-17A expression (boxed) in naive CD4^+^ T cells from WT, *Sumo1*^−*/*−^ (top), and *Sumo3*^−*/*−^ (bottom) mice, cultured in vitro for 3 d under T_H_17 priming conditions. Numbers adjacent to the outlined area indicate the percentage of the cells in gated area (throughout). **b** qPCR analysis of *Il17a*, *Il17f*, *Il22*, *Ccr6*, *Ccl20*, and *Ahr* mRNA in WT and *Sumo3*^*−/*−^ T_H_17 cells assessed in (**a**). Expression is presented relative to that of the control gene *Actb*. **c** Mean clinical EAE scores of *Rag1*^*−/*−^ mice adoptively transferred with WT or *Sumo3*^−*/*−^ CD4^+^ T cells (key; *n* *=* *5* per genotype) from days 0 to 35 after immunization with the EAE-inducing epitope MOG_35-55_. **d** Quantification of CNS-infiltrating cells from *Rag1*^−/−^ mice reconstituted with CD4^+^ T cells from WT or  *Sumo3*^−/−^mice (same as in **c**) expressing characteristic mononuclear cell surface markers, assessed using flow cytometry at the peak of disease. **e** Flow cytometric analysis of CNS-infiltrating cells from *Rag1*^−/−^ mice reconsituted with WT or *Sumo3*^−/−^ CD4^+^ T cells (same as in **c**) positive for intracellular cytokines IL-17A^+^, IFNγ^+^, GM^-^CSF^+^, IL-17A^+^ IFNγ^+^, and IL-17A^+^ GM^-^CSF^+^. NS, not significant (*P* > 0.05); *P < 0.05 (*t-*test); **P < 0.01 (*t-*test). Data are from three experiments (**a**, right; and **b**–**e**; presented as median [central line], maximum and minimum [box ends], and outliers [extended lines]) or are from one representative of three independent experiments (**a**, left)

To investigate whether sumoylation plays a role in T helper cell differentiation, we examined the differentiation of *Sumo1*^−/−^ and *Sumo3*^*-−/*−^ CD4^+^ T lymphocytes (*Sumo2*^−*/*−^ mice are embryonic lethal^31^). Deletion of *Sumo1* compromised T_H_1 and Treg differentiation, but did not affect T_H_2 differentiation (Supplementary Fig. 1a). Deletion of *Sumo3*, but not *Sumo1*, dramatically impaired T_H_17 differentiation (Fig. 1a) and decreased expression of critical T_H_17 genes (Fig. 1b). However, *Sumo3*^*−/−*^ CD4^+^ T cells could normally differentiate into T_H_1, T_H_2, and Treg lineages (Supplementary Fig. 1b). We next adoptively transferred *Sumo3*^−*/−*^ CD4^+^ T cells into *Rag1*^−*/*−^ mice to test their ability to induce EAE. *Rag1*^−*/*−^ mice reconstituted with *Sumo3*^−*/−*^ CD4^+^ T cells had attenuated disease severity (Fig. 1c), which correlated with lower infiltration of lymphocytes, including Ly6G^+^ neutrophils, CD4^+^ T cells, and CD11b^+^Ly6C^+^ monocytes, into the central nervous system (CNS; Fig. 1d and Supplementary Fig.1c for gating strategy). In addition, the percentages (Supplementary Fig.1d) and numbers (Fig. 1e) of CNS-infiltrating IL-17A^+^, IFNγ^+^, GM-CSF^+^, IL-17A^+^IFNγ^+^, and IL-17A^+^GM-CSF^+^ CD4^+^ T cells responsible for EAE were also significantly lower in these mice^7–9^. These results suggest that SUMO3, but not SUMO1, promotes RORγt-dependent T_H_17 differentiation.

### *Sumo3*, but not *Sumo1*, is required for thymic ISP progression

**Fig. 2:**
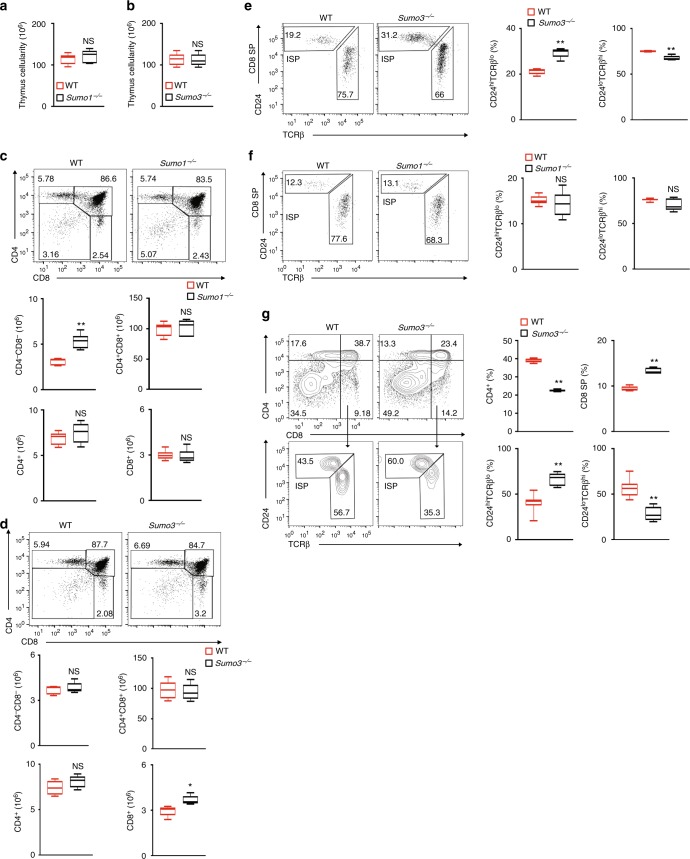
SUMO3, but not SUMO1, is required for the progression of thymic ISPs. **a**, **b** Thymic cellularity of WT and **a** *Sumo1*^−*/*−^or **b** *Sumo3*^−*/*−^ mice (*n* *=* *5* per genotype). **c**, **d** Representative flow cytometric analysis of CD4 and CD8 on the surface of thymocytes from WT and **c** *Sumo1*^−*/*−^ or **d** *Sumo3*^−*/*−^ mice (top two panels). The bottom panels present the absolute numbers of CD4^+^, CD8^+^, CD4^−^CD8^−^, and CD4^+^CD8^+^ thymocytes for individual mice (*n* = 5 per genotype). **e**, **f** Representative flow cytometric analysis of CD24 and TCRβ expression in CD8^+^ cells of WT and **e** *Sumo3*^−/−^ or **f** *Sumo1*^−*/*−^ thymi (two panels on the left). The two panels on right present the percentages of immature TCR^lo^CD24^hi^ ISPs and mature TCR^hi^CD24^lo^ cells in the thymi of individual mice (*n* *=* *5* per genotype). **g** Representative flow cytometric analysis of CD4 and CD8 expression in cells differentiated from sorted WT and *Sumo3*^*−/*^^−^ CD4^-^CD8^−^thymocytes co-cultured for 3 d with OP9-DL4 stroma cells and IL-7 (5 ng/ml) to assess ex vivo thymocyte development (top two panels on the left). The top two panels on the right present the percentages of CD4^+^ and CD8^+^ cells differentiated from individual mice (*n* *=* *5* per genotype). The bottom two panels on the left show flow cytometric analysis of CD24 and TCRβ expression in CD8^+^ cells from the top panels. The bottom two panels on the right present the percentages of immature TCR^lo^CD24^hi^ ISPs and mature TCR^hi^CD24^lo^ thymocytes from individual mice (*n* *=* *5* per genotype). NS, not significant (*P* > 0.05); *P < 0.05 (*t-*test); **P < 0.01 (*t-*test). Data are from three experiments (**a**, **b**; **c**, **d**, four bottom panels; **e**–**g**, two right panels; presented as median [central line], maximum and minimum [box ends], and outliers [extended lines]) or are from one representative of three independent experiments (**c**, **d**, top two panels; **e**–**g**, two panels on left)

To determine whether sumoylation plays a role in RORγt-dependent thymocyte development, we analyzed thymocytes from *Sumo3*^*−/*−^ and *Sumo1*^*−/*−^ mice. The thymic cellularity of *Sumo1*^*−/*−^ (Fig. 2a) and *Sumo3*^*−/*−^ (Fig. 2b) mice was equivalent to that of wild-type (WT) mice. We then analyzed CD4 and CD8 markers to monitor the three sequential stages of thymocyte development: CD4^−^CD8^−^ double-negative (DN), CD4^+^CD8^+^ double-positive (DP), and CD4^+^ or CD8^+^ single-positive (SP). We did not detect obvious differences in the overall percentages of DN, DP, and SP populations in the thymi of WT versus *Sumo1*^*−/*−^ mice (Fig. 2c). We did, however, notice an increased percentage of CD8^+^ SP cells in the thymi of *Sumo3*^*−/*−^ mice (Fig. 2d). Further scrutiny of the CD8^+^ SP population revealed a significantly greater percentage of immature TCR^lo^CD24^hi^CD8^+^ cells (ISPs), as well as a correspondingly lower percentage of mature TCR^hi^CD24^lo^CD8^+^ cells, in the thymi of *Sumo3*^*−/*−^ (Fig. 2e), but not *Sumo1*^*−/*−^ (Fig. 2f), mice. These findings indicate the selective function of *Sumo3* in the progression of ISP, which is RORγt-dependent^18^. Furthermore, whereas the absolute number of ISPs was increased in *Sumo3*^*−/*−^ compared to WT thymi (Supplementary Fig. 1e), there was no difference in the number of mature TCR^hi^CD24^lo^CD8^+^ cells in WT and *Sumo3*^*−/*−^ thymi, suggesting that the overall increase in CD8^+^ SP cells observed in *Sumo3*^*−/−*^ thymi is due to increased ISPs and not mature CD8^+^ cells. To determine the intrinsic function of SUMO3 in thymocyte development, we isolated and co-cultured CD4^−^CD8^−^ DN thymocytes with OP9-DL4 stroma cells to observe their differentiation in vitro^32^ (Fig. 2g). Although both WT and *Sumo3*^*−/*−^ DN cells could differentiate into DP and SP populations, there were increased percentages and numbers of CD8^+^ SP but not CD4^+^ SP cells in *Sumo3*^*−/−*^ cultures (Fig. 2g, top panels). Furthermore, we detected significantly more TCR^lo^CD24^hi^CD8^+^ ISPs among *Sumo3*^*−/*−^ CD8^+^ cells than among WT CD8^+^ cells (Fig. 2g, bottom panels), suggesting the intrinsic requirement of SUMO3 for the progression of ISPs. We previously found that, similarly to the deletion of *Sumo3* shown here, the deletion of RORγt in mice resulted in more ISPs and reduced T_H_17 differentiation^33^, which suggested that RORγt may be SUMO3-modified.

### Sumoylation of K31 is essential for RORγt function

**Fig. 3:**
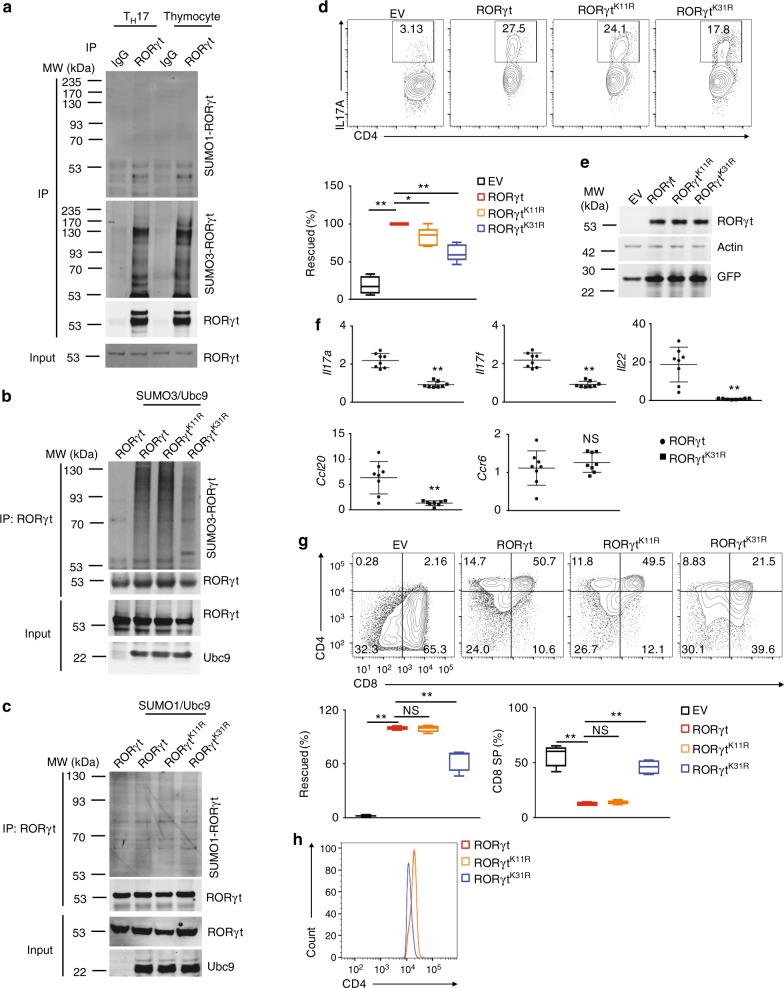
K31 sumoylation is essential for RORγt to regulate T_H_17 and thymocyte differentiation. **a** Immunoblot analysis of SUMO1- or SUMO3-modified RORγt among proteins immunoprecipitated using indicated antibodies in differentiated T_H_17 cells or thymocytes. The bottom panel shows the immunoblot analysis of total RORγt, used as a loading control throughout. Molecular weights in kilodaltons (kDa) are shown on the left. **b**, **c** Immunoblot analysis of **b** SUMO3- or **c** SUMO1-modified RORγt immunoprecipitated from HEK293 T cells expressing indicated proteins. **d** Representative flow cytometric analysis of IL-17A^+^ cells (boxed) among *Rorγt*^*−/*−^ CD4^+^ T cells transduced with retroviruses expressing GFP alone (EV) or GFP with indicated RORγt, polarized for 3 d under T_H_17-priming conditions. The bottom panel presents the percentages of IL-17A^+^ cells rescued by retroviral transduction in independent samples (*n* *=* *8* per group). 100% represents the number of IL-17A^+^ cells after transduction with WT RORγt. **e** Immunoblot analysis of indicated proteins in differentiated T_H_17 cells shown in **d**. **f** qPCR analysis of indicated gene expression in the T_H_17 cells shown in **d**. Expression is presented relative to that of the control gene *Actb*. **g** Representative flow cytometric analysis of CD4 and CD8 expression in cells differentiated from *Rorγt*^*−/*−^ CD4^−^CD8^−^thymocytes transduced with retroviruses, as described in **d**, and co-cultured for 3 d with OP9-DL4 cells (top four panels). The left panel in the second row presents the percentages by which thymocyte development was rescued by retroviral transduction in independent samples (*n* *=* *8* per group). 100% represents the number of thymocytes after transduction with WT RORγt. The right panel in the second row presents the percentages of CD8^+^ cells in independent samples (*n* *=* *8* per group). **h** Representative flow cytometric analysis of CD4 expression among the CD4^+^CD8^+^ thymocytes assessed in **g**. NS, not significant (*P* > 0.05); *P < 0.05 (*t-t*est); **P < 0.01 (*t-*test). Data are from three experiments (**d**, bottom panel; **e**; **g**, bottom panels; presented as median [central line], maximum and minimum [box ends], and outliers [extended lines]) or are from one representative of three independent experiments (**a**–**c**; **d**, top panels; **f**; **g**, top panels; **h**)

To determine whether RORγt is sumoylated, we monitored the sumoylation of immunoprecipitated RORγt using anti-SUMO1 and anti-SUMO3 antibodies (Fig. 3a, and Supplementary Fig 9 for the full-length image of immunoblot). Whereas SUMO1-modified RORγt (SUMO1-RORγt) was barely detectable (Fig. 3a, top panel), SUMO3-modified RORγt (SUMO3-RORγt) produced strong signals in both T_H_17 cells and thymocytes (Fig. 3a, second panel). To identify the sumoylated residues, immunoprecipitated RORγt was subject to mass spectrometric analysis to detect a signature peptide containing a “QTGG” remnant at the sumoylation site. Lysines 11 and 31 (K11 and K31) were identified as the sumoylation sites in RORγt (Supplementary Fig. 2a). To confirm these sites, K11 and K31 were mutated to arginine to prevent the sumoylation, and sumoylation of purified WT and mutant RORγt was compared in vitro (Fig. 3b). As expected, we observed that SUMO3-RORγt could not be detected in the absence of the E2 enzyme Ubc9 or SUMO3. We also observed that, whereas the K11R mutation (RORγt^K11R^) did not affect SUMO3-RORγt levels, the K31R mutation (RORγt^K11R^) greatly reduced SUMO3-RORγt (Fig. 3b). In contrast, we could not detect obvious SUMO1-modified RORγt, RORγt^K11R^, or RORγt^K31R^ (Fig. 3c). These results strongly suggest that RORγt is SUMO3- but not SUMO1-modified at K31. Interestingly, sequence alignment indicated that K31 and its surrounding amino acid sequence are highly conserved in RORγt across species (Supplementary Fig. 2b), suggesting the importance of K31 as a sumoylation site.

To determine the role of K31 sumoylation in RORγt-dependent functions, we compared the ability of retrovirally expressed RORγt and RORγt^K31R^ to rescue T_H_17 differentiation in *Rorγt*^−*/−*^ CD4^+^ T cells (Fig. 3d). As expected, *Rorγt*^*−/*^^−^ CD4^+^ T cells transduced with retroviruses expressing GFP alone (empty virus, EV) failed to differentiate into T_H_17 cells. T_H_17 cell differentiation was rescued by WT RORγt and RORγt^K11R^, but not RORγt^K31R^ (Fig. 3d, and Supplementary Fig. 2c for gating strategy), although both mutants were expressed at the levels comparable to WT RORγt expression (Fig. 3e). Consistent with these results, the expression of critical T_H_17 genes was lower in RORγt^K31R^-reconstituted *Rorγt*^*−/−*^ T cells than in WT RORγt-reconstituted T cells (Fig. 3f), confirming that the T_H_17 differentiation program is impaired when K31 sumoylation is blocked.

To determine whether K31 sumoylation is essential for RORγt-regulated thymocyte development, we compared the development of *Rorγt*^*−/−*^ thymocytes retrovirally reconstituted with RORγt, RORγt^K11R^, and RORγt^K31R^ in vitro (Fig. 3g, and Supplementary Fig. 2d for gating strategy). Isolated *Rorγt*^*−/−*^ CD4^−^CD8^−^ DN thymocytes transduced with retroviruses simultaneously expressing GFP and RORγt or RORγt^K11R^, but not expressing GFP alone (EV), differentiated into CD4^+^CD8^+^ DP and CD4^+^ SP cells. However, retroviral expression of RORγt^K31R^ failed to fully restore thymocyte development, indicated by more CD4^−^CD8^−^ DN and CD8^+^ SP cells and fewer CD4^+^CD8^+^ DP and CD4^+^ SP cells (Fig. 3g). Interestingly, the expression of surface CD4, which is lower in *Rorγt*^*−/−*^ thymocytes than in WT thymocytes^18^, was rescued in *Rorγt*^*−/−*^ cells reconstituted with WT RORγt or RORγt^K11R^ but not with RORγt^K31R^ (Fig. 3h), suggesting a role of K31 sumoylation in the regulation of CD4 expression. Altogether, these data demonstrate that blocking sumoylation at K31 impairs RORγt functions in thymocyte development and T_H_17 differentiation in vitro.

### *RORγt*^*K31R/K31R*^ mice exhibit defective T_H_17 differentiation

**Fig. 4:**
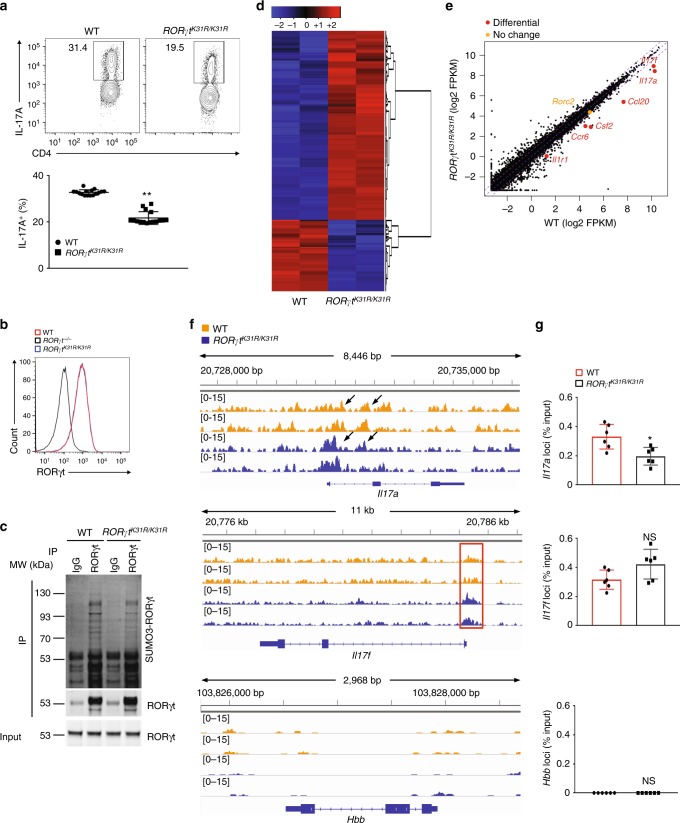
CD4^+^ T cells from *RORγt*^*K31R/K31R*^ mice exhibit defective T_H_17 differentiation. **a** Representative flow cytometric analysis of the percentages of IL-17A^+^ cells (boxed) among WT or *RORγt*^*K31R/K31R*^ CD4^+^ T cells polarized for 3 d under T_H_17-priming conditions. The bottom panel presents the percentages of IL-17A^+^ cells in independent samples. **b** Representative flow cytometric analysis of RORγt expression among CD4^+^ cells shown in **a** and their *Rorγt*^*−/−*^ counterpart. **c** Immunoblot analysis of SUMO3-modified RORγt immunoprecipitated using IgG or anti-RORγt antibodies from WT or *RORγt*^*K31R/K31R*^ CD4^+^ cells polarized under T_H_17 conditions. **d** RNA-seq analysis of genes (one per row) upregulated (red) or downregulated (blue) in the WT or *RORγt*^*K31R/K31R*^ CD4^+^ cells assessed in **a**. Two biological replicates, one per column, are shown for each genotype. Expression of each gene is presented relative to its average expression across all samples. **e** Comparison of the gene expression profile of the WT and *RORγt*^*K31R/K31R*^ cells assessed in **a**, presented as fragments per kilobase of transcript per million mapped reads (FRKM). The colors indicate genes encoding molecules critical for T_H_17 cells that are downregulated (red) or comparably expressed (orange) in *RORγt*^*K31R/K31R*^ cells compared to WT cells. **f** ChIP-seq analysis identified RORγt DNA-binding peaks (delineated by a red rectangle) in *Il17a* (top), *Il17f* (middle), and negative control *Hbb* (bottom) in the WT (yellow) and *RORγt*^*K31R/K31R*^ (blue) cells assessed in **d** (two biological replicates of each; one per line). **g** ChIP analysis of RORγt binding to *Il17a* (top), *Il17f* (middle), and *Hbb* (bottom) in the cells assessed in **a**. NS, not significant (*P* > 0.05); *P < 0.05 (*t-*test); **P < 0.01 (*t-*test). Data are from three experiments (**a**, bottom panel), two experiments (**g**; mean ± s.e.m), or are one representative of three independent experiments (**a**, top panel; **b**; **c**; **f**)

To investigate the function of K31 sumoylation in vivo, we generated a strain of mouse expressing RORγt^K31R^ (*RORγt*^*K31R*/*K31R*^) (Supplementary Fig. 3a–3c). The number of splenocytes was slightly higher in *RORγ*^*tK31R/K31R*^ mice than in WT mice, which was partially attributed to increased CD8^+^ but not CD4^+^ T cells (Supplementary Fig. 4a). T cells from *RORγt*^*K31R/K31R*^ mice consistently exhibited defective T_H_17 differentiation, as indicated by the lower generation of IL-17A^+^ cells compared to WT mice (Fig. 4a) and decreased expression of critical T_H_17 genes (Supplementary Fig. 4b). However, the T cells from *RORγt*^*K31R/K31R*^ mice differentiated into T_H_1, T_H_2, and Treg comparably to T cells from WT mice (Supplementary Fig. 4c), suggesting a selective defect in differentiation into T_H_17 cells. The observed reduction in T_H_17 differentiation was not due to decreased expression of RORγt^K31R^, which was comparable with WT RORγt expression in T_H_17 cells (Fig. 4b). To confirm that K31R mutation affects the sumoylation of RORγt in vivo, we compared levels of SUMO3- RORγt in differentiated T_H_17 cells from WT and *RORγt*^*K31R*/*K31R*^ mice. Indeed, SUMO3-modified (Fig. 4c), but not ubiquitin-modified (Supplementary Fig. 4d), RORγt was significantly reduced in *RORγt*^*K31R*^ T_H_17 cells, confirming that RORγt-K31 is sumoylated in vivo.

To assess the global effects of K31 sumoylation on T_H_17 differentiation, we mapped RORγt DNA-binding sites using ChIP-seq and gene expression profiles using RNA-seq in WT and *RORγt*^*K31R*/*K31R*^ T_H_17 cells. We found similar expression patterns in biological replicates of WT and *RORγt*^*K31R/K31R*^ T_H_17 cells, as indicated by the heat map in Fig. 4d showing similarly up and downregulated genes. Many T_H_17 genes, including *Il17a, Il17f, Ccl20, Csf2, Ccr6*, and *Il1r1*, but not *Rorc*, were down-regulated in *RORγt*^*K31R*/*K31R*^ T_H_17 cells (Fig. 4e), suggesting an essential function of RORγt K31 sumoylation in the expression of genes critical for T_H_17 differentiation. ChIP-seq analysis identified DNA-binding peaks within critical T_H_17 gene loci, including *Il17a* and *Il17f*, which overlap well with our previously identified RORγt DNA-binding peaks (Supplementary Fig. 4e)^33^. Furthermore, we conducted a search among all the RORγt DNA-binding peaks to identify potential transcription factor binding motifs, and the most enriched motif was the RORγt binding site in both WT and *RORγt*^*K31R*/*K31R*^ T_H_17 cells (Supplementary Fig. 4f), validating the results of our ChIP-seq assay. We more carefully compared the RORγt DNA-binding peaks at the IL-17 loci in WT and *RORγt*^*K31R*/*K31R*^ T_H_17 cells (Fig. 4f, top two panels), using the *Hbb* locus as a negative control (Fig. 4f, bottom panel). Some *RORγt*^*K31R*^ DNA-binding peaks were smaller than those of WT RORγt at the *Il17a* locus (Fig. 4f), indicating that reduced *RORγt*^*K31R*^ DNA-binding affinity likely contributes to the reduced expression of *Il17a* observed in *RORγt*^*K31R*^ T_H_17 cells. On the other hand, the *RORγt*^*K31R*^ DNA-binding peaks at the *IL17f* locus were just as great as, if not greater than, those of WT RORγt, which suggests that sumoylation of RORγt may stimulate the expression of *Il17f* via DNA-binding-independent mechanisms. These findings were further confirmed using individual ChIP assays (Fig. 4g). Taken together, our results demonstrate that K31 sumoylation of RORγt promotes T_H_17 differentiation by activating the expression of the critical T_H_17 genes.

### *RORγt*^*K31R/K31R*^ mice are resistant to induction of EAE

**Fig. 5:**
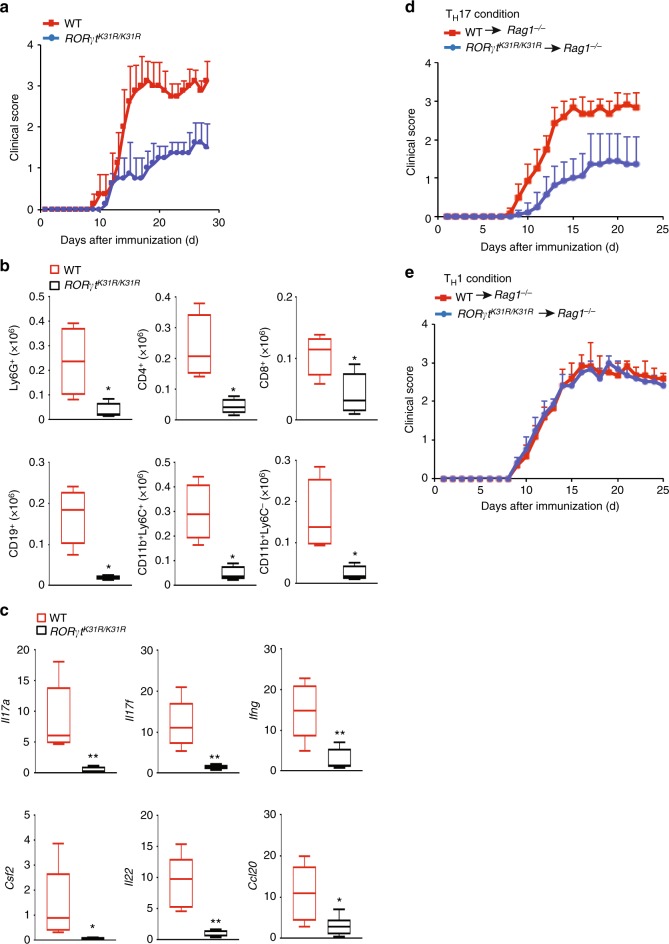
*RORγt*^*K31R/K31R*^ mice are resistant to induction of EAE. **a** Mean clinical EAE scores of female WT and *RORγt*^*K31R/K31R*^ mice (*n* *=* *10* per genotype) from days 0 to 30 after immunization with the EAE-inducing epitope MOG_35-55_. **b** Quantification of CNS-infiltrating cells from WT and *RORγt*^*K31R/K31R*^ mice in which EAE was induced (same as in **a**) expressing characteristic mononuclear cell surface markers, assessed using flow cytometry at the peak of disease. **c** qPCR analysis of cytokine-encoding *Il17a* (top left), *Il17f* (top middle), *Ifng* (top right), *Csf2* (bottom left), *Il22* (bottom middle) and *Ccl20* (bottom right) mRNA in the CNS-infiltrating lymphocytes assessed in **a**. Expression is presented relative to that of the control gene *Actb*. **d** Mean clinical EAE scores of female *Rag1*^*−/−*^ mice reconstituted with CD4^+^ T cells from MOG_35-55_-primed WT or *RORγt*^*K31R/K31R*^ mice (*n* *=* *5* per genotype) that were further expanded in vitro for 3 d in the presence of MOG_35–55_ and IL-23 (20 ng/ml) (T_H_17 conditions). **e** Mean clinical EAE scores of female *Rag1*^*−/−*^ mice reconstituted with CD4^+^ T cells from MOG_35–55_-primed WT or *RORγt*^*K31R/K31R*^ mice (*n* *=* *5* per genotype) that were further expanded in vitro for 3 d in the presence of MOG_35-55_ and IL-12 (20 ng/ml) (T_H_1 conditions). *P < 0.05 (*t-*test); **P < 0.01 (*t-*test). Data are from three experiments (**b**; **c**, presented as median [central line], maximum and minimum [box ends], and outliers [extended lines])

To determine the function of sumoylation in RORγt-dependent immunity in vivo, we induced EAE in WT and *RORγt*^*K31R/K31R*^ mice. The severity of the disease was markedly attenuated in *RORγt*^*K31R/K31R*^ mice compared with WT mice (Fig. 5a), which was reflected in lower CNS infiltration by various mononuclear cells (Fig. 5b), indicating reduced inflammation. In addition, there was lower expression of critical T_H_17 genes in CNS-infiltrating lymphocytes recovered from *RORγt*^*K31R/K31R*^ mice than in those recovered from WT mice (Fig. 5c). Therefore, we have demonstrated that sumoylation of RORγt-K31 modulates T_H_17-mediated EAE in vivo.

Because both T_H_17 and T_H_1 cells can induce EAE^34^, we compared the ability of T_H_1 and T_H_17 cells derived from *RORγt*^*K31R*/*K31R*^ and WT mice to induce passive EAE. For this purpose, T cells from WT or *RORγt*^*K31R*/*K31R*^ mice primed with myelin oligodendrocyte glycoprotein 35–55 (MOG_35–55_) were cultured and stimulated with MOG_35–55_ in vitro under T_H_17- or T_H_1-polarizing conditions, and then adoptively transferred to *Rag1*^*−/−*^ mice to induce EAE. Compared to their WT counterparts, *RORγt*^*K31R*/*K31R*^ T cells that were expanded under T_H_17 conditions induced less severe EAE (Fig. 5d), which was associated with lower CNS infiltration by various mononuclear lymphocytes (Supplementary Fig. 5a) and lower expression of critical T_H_17 genes in lymphocytes recovered from the CNS (Supplementary Fig. 5b). In contrast, *RORγt*^*K31R/K31R*^ and WT T cells stimulated under T_H_1 conditions did not differentially induce EAE in *Rag1*^*−/−*^ mice (Fig. 5e), which was demonstrated by mostly comparable numbers of various CNS-infiltrating mononuclear lymphocytes (Supplementary Fig. 5c). These results indicate that sumoylation at K31 is required selectively for RORγt-dependent T_H_17 immunity in vivo.

### ISPs accumulate in thymi of *RORγt*^*K31R/K31R*^ mice

**Fig. 6:**
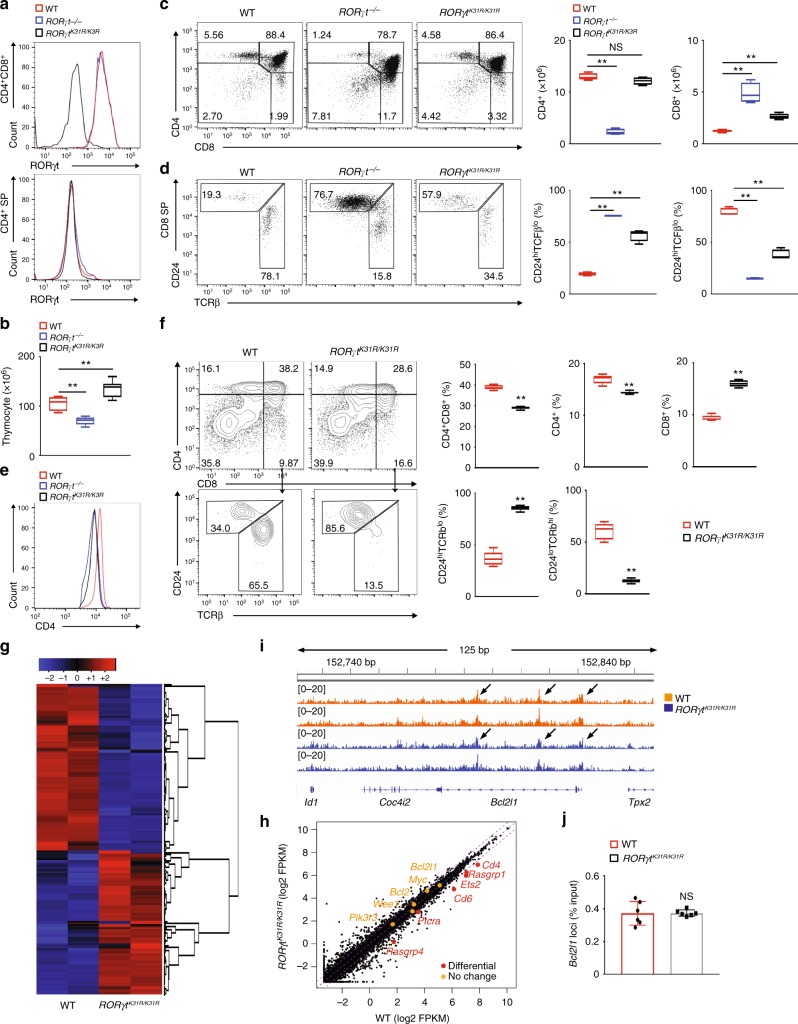
ISPs accumulate in *RORγt*^*K31R/K31R*^ thymi. **a** Flow cytometric analysis of ROR*γ*t in the CD4^+^ or CD4^+^CD8^+^ thymocytes of indicated mice. **b** Thymic cellularity of indicated mice (*n* *=* *5*). **c** Cytometric analysis of CD4 and CD8 expression in thymocytes of indicated mice (three panels on left). The two panels on the right present the numbers of CD4^+^ and CD8^+^ cells among thymocytes from individual mice (*n* *=* *5*). **d** Flow cytometric analysis of CD24 and TCRβ expression among CD8^+^ cells shown in (**c**) (three panels on left). The two panels on the right present the frequency of indicated cells among the thymocytes (*n* *=* *5*). **e** Flow cytometric analysis of CD4 levels among CD4^+^CD8^+^ thymocytes. **f** Flow cytometric analysis of CD4 and CD8 expression on in vitro differentiated thymocytes of the indicated mice (top two panels on the left). The top three panels on the right present the percentages of indicated cells differentiated in vitro (*n* *=* *5*). The bottom panels on the left present the cytometric analysis of CD24 and TCRβ expression in the CD8^+^ subpopulation from the top panels. The bottom two panels on the right present the percentages of indicated thymocytes among the CD8^+^ cells (*n* *=* *6*). **g** RNA-seq analysis of genes upregulated (red) or downregulated (blue) in thymocytes of the indicated mice assessed in **f**. Two biological replicates each genotype. **h** Comparison of the gene expression profile of the thymocytes assessed in **g**. The colors indicate downregulated (red) or comparably expressed genes (orange) in *RORγt*^*K31R/K31R*^ compared to WT thymocytes. **i** ChIP-seq analysis identified RORγt DNA-binding peaks (arrows) in*Bcl2l1* in the cells assessed in **g** (two biological replicates). **j** ChIP-qPCR analysis of RORγt binding to*Bcl2l1 *in the thymocytes assessed in **f**. NS, not significant (*P* > 0.05); **P < 0.01 (*t-*test). Data are from three experiments (**b**; **c**, **d**, two panels on the right; **f**, right panels; **j**; presented as median [central line], maximum and minimum [box ends], and outliers [extended lines]), are pooled from two biological replicates (**g**, **h**), or are one representative of three independent experiments (**a**; **c**, **d**, left; **e**; **i**)

To determine the function of K31 RORγt sumoylation in thymocyte development, we analyzed thymocytes from WT, *RORγt*^*−/−*^, and *RORγt*^*K31R*/*K31R*^ mice. The expression of RORγt^K31R^ and WT RORγt was equivalent in CD4^+^CD8^+^ thymocytes (Fig. 6a, top panel) and non-detectable in CD4^+^ SP cells (Fig.6a, bottom panel), suggesting that K31R does not disturb the expression pattern of RORγt. However, SUMO3-modified (Supplementary Fig. 6a), but not ubiquitinated (Supplementary 6b), RORγt was lower in *RORγt*^*K31R/K31R*^ thymocytes compared to those of WT thymocytes, confirming K31 as an RORγt sumoylation site in thymocytes. RORγt is known to regulate the survival and cell cycle of thymocytes by upregulating the expression of Bcl-x_L_^18^. However, WT and *RORγt*^*K31R/K31R*^ mice had comparable thymocyte survival (Supplementary Fig. 6c) and percentages of cells with > 2 N of DNA (in the DNA synthesis phase) (Supplementary Fig. 6d). In addition, *RORγt*^*K31R/K31R*^ mice had lower rather than greater thymic cellularity that WT mice (Fig. 6b). These results suggest that K31 sumoylation is dispensable for RORγt-dependent thymocyte survival and cell cycle regulation. Analysis of the surface markers CD4 and CD8 revealed greater percentages of CD4^−^CD8^−^ DN and CD8^+^ SP cells in *RORγt*^*K31R/K31R*^ thymi than in WT thymi, similar to those observed in *Rorγt*^*−/−*^ thymi (Fig. 6c, three panels on the left, and Supplementary Fig. 6e). In addition, the absolute number of CD8^+^ SP but not CD4^+^ SP cells (Fig. 6c, two panels on right) was greater in *RORγt*^*K31R/K31R*^ thymi than in WT. Among CD8^+^ SP cells, there was a higher frequency of immature TCR^lo^CD24^hi^CD8^+^ cells (ISPs) in *RORγt*^*K31R/K31R*^ thymi, similar to the frequency in *Rorγt*^*−/−*^ thymi (Fig. 6d), as well as *Sumo3*^*−/−*^ thymi (Fig. 2e). Not only the frequency but the absolute number of ISPs was greater in *RORγt*^*K31R/K31R*^ thymi compared to WT thymi, whereas the cellularity of mature TCR^hi^CD24^lo^CD8^+^ cells was comparable between groups (Supplementary Fig. 6f). These results indicate the critical function of RORγt-K31 sumoylation in the progression of ISPs. In addition, we observed lower levels of surface CD4 on CD4^+^CD8^+^ DP cells in both *RORγt*^*K31R/K31R*^ and *Rorγt*^*−/−*^ thymi compared to WT thymi (Fig. 6e), suggesting a positive role of RORγt-K31 sumoylation in CD4 expression. We next compared the differentiation of sorted WT and *RORγt*^*K31R*/*K31R*^ CD4^−^CD8^−^ DN thymic cells co-cultured with stroma cells in vitro (Fig. 6f). As expected, DN cells derived from *RORγt*^*K31R*/*K31R*^ mice gave rise to a higher frequency of CD8^+^ SP cells and a greater percentage of TCR^lo^CD24^hi^CD8^+^ ISPs than DN cells from WT mice. We thus separated the functions of RORγt in thymocyte development into two categories: (1) K31 sumoylation-independent functions, including survival and cell cycle, and 2) K31 sumoylation-dependent functions, including the progression of ISPs and CD4 expression.

To better understand the K31 sumoylation-dependent and -independent functions of RORγt, we mapped the landscape of RORγt DNA-binding sites and examined the gene expression profiles of WT and *RORγt*^*K31R*/*K31R*^ thymocytes using ChIP-seq and RNA-seq assays. We observed similar expression patterns among biological replicates of WT or *RORγt*^*K31R/K31R*^ thymocytes, as suggested by the color patterns in the clustered heat map shown in Fig. 6g, which demonstrates the reproducibility of our RNA-seq assay. We previously showed that the reduced survival and dysregulated cell cycle of *Rorγt*^*−/−*^ thymocytes were associated with significant changes in the expression of several important cell survival and cell cycle regulators^33^. The expression of several of these regulators, *Pik3r3, Wee1, Bcl2, Myc*, and *Bcl2l1*, was not significantly different between WT and *RORγt*^*K31R/K31R*^ thymocytes (Fig. 6h, genes listed on the left in orange). Therefore, K31 sumoylation does not appear to be required for the expression of these critical survival and cell cycle regulators, which explains why thymocyte survival and cell cycle regulation are K31 sumoylation-independent. However, we identified several genes, including *Rasgrp1*, Ets2, *Cd6*, *Ptcra*, *Rasgrp4*, and *Cd4*, that were downregulated in *RORγt*^*K31R/K31R*^ thymocytes compared to WT thymocytes (Fig. 6h, genes listed on the right in red) and are known to regulate thymocyte development^35–38^. We found that the CD4^−^encoding *Cd4* gene was downregulated in *RORγt*^*K31R*/*K31R*^ thymocytes compared to WT thymocytes (Fig. 6h, top gene in red), which is consistent with lower protein levels of CD4 on *RORγt*^*K31R/K31R*^ thymocytes (Fig. 6e), as well as *Rorγt*^*−/−*^ thymocytes retrovirally reconstituted with RORγt^K31R^ (Fig. 3h). In particular, *Cd6* was reported to regulate the progression of ISPs^35^. Therefore, we have demonstrated that RORγt K31 sumoylation is required for the transactivation of these genes, which are likely responsible for K31 sumoylation-dependent functions, such as the progression of ISPs. Our ChIP-seq assay also identified obvious RORγt DNA-binding peaks at *Bcl2l1*(Fig. 6i, j)*, Cd6, Ets2, Rasgrp1*, and *Cd4* loci (Supplementary Fig. 6g), suggesting that they are direct targets of RORγt. Interestingly, RORγt^K31R^ binds to the same sites on these gene loci as WT RORγt, suggesting that K31 sumoylation is not required for the DNA binding of RORγt. Therefore, K31 sumoylation of RORγt likely regulates the expression of these target genes through DNA binding-independent mechanisms.

RORγt is required for the development of secondary lymph tissues^18^. To determine the roles of K31 sumoylation in RORγt-dependent organogenesis, we examined lymph tissues in *RORγt*^*K31R*/*K31R*^ and WT mice. *RORγt*^*K31R/K31R*^ mice had all the lymph nodes observed in WT mice except for Peyer’s patches (Supplementary Fig. 6h), suggesting a selective role of K31 sumoylation in the biogenesis of Peyer’s patches.

### Sumoylation stabilizes RORγt–KAT2A–SRC1 complexes

**Fig. 7:**
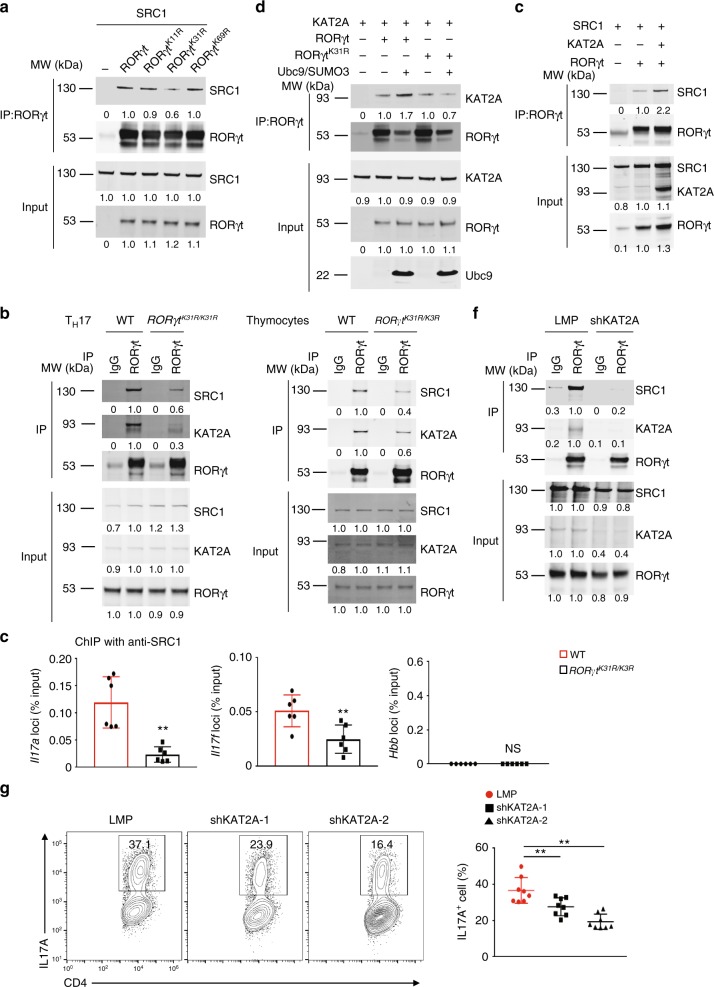
Sumoylation of RORγt-K31 stimulates the recruitment of KAT2A and co-activator SRC1. **a** Immunoblot analysis of SRC1 among immunoprecipitated RORγt from HEK293T cells co-transfected with plasmids to express SRC1 and WT or mutant (K11R, K31R, and K69R) RORγt (top blots). The bottom plots throughout the figure show the immunoblot analysis of whole-cell lysates without immunoprecipitation (input). The numbers under the blots throughout the figure represent the quantified expression, relative to that in WT RORγt samples, determined by density. **b** Immunoblot analysis of KAT2A and SRC1 among immunoprecipitated proteins (using IgG or anti-RORγt antibodies, as indicated) from WT or *RORγt*^*K31R/K31R*^ CD4^+^ T cells polarized under T_H_17 conditions in vitro (left panel) or thymocytes (right panel). **c** ChIP analysis of SRC1 binding to *Il17a* (left), *Il17f* (middle), and *Hbb* (right) in WT or *ROR*γ*t*^*K31R/K31R*^ CD4^+^ T cells polarized under T_H_17 conditions. **d** Immunoblot analysis of KAT2A among immunoprecipitated RORγt from HEK293T cells co-transfected plasmids to express various combinations (above lanes) of Ubc9, SUMO3, KAT2A, and RORγt or RORγt ^K31R^ (top blots). **e** Immunoblot analysis of SRC1 among immunoprecipitated RORγt from HEK293T cells co-transfected with plasmids to express various combinations of SRC1, KAT2A, and RORγt. **f** Immunoblot analysis of SRC1 and KAT2A among immunoprecipitated proteins (using IgG or anti-RORγt antibodies, as indicated) from WT CD4^+^ T cells transduced with retroviruses expressing GFP alone (LMP) or GFP with small hairpin RNA targeting KAT2A (shKAT2A) and polarized for 3 d under T_H_17-priming conditions. **g** Representative flow cytometric analysis of the percentage of IL-17A^+^ cells (boxed) among WT CD4^+^ T cells transduced with retroviruses expressing GFP alone (LMP) or GFP with shKAT2A and polarized for 3 d under T_H_17-priming conditions (left). The panel on the right presents the percentages of IL-17A^+^ cells among CD4^+^ cells from independent samples. NS, not significant (*P* > 0.05); **P < 0.01 (*t*-test). Data are from three experiments (**c**; **g**, right panel; mean ± s.e.m), or are one representative of three independent experiments (**a**, **b**; **d**–**f**; **g**, left panels)

One function of sumoylation is to regulate protein stability^39^. However, this does not seem to be the case for K31 sumoylation of RORγt, as the protein levels of RORγt and RORγt^K31R^ in both T_H_17 cells (Fig. 4b) and thymocytes (Fig. 6a) were equivalent and the degradation rates of RORγt and RORγt^K31R^ were comparable (Supplementary Fig. 7a). Given that sumoylation can also regulate protein–protein interactions by adding a new docking site, we tested whether K31 sumoylation regulated the binding of RORγt to its co-factors. RORγt is known to interact with co-activator SRC1 to regulate T_H_17 differentiation^25^. Indeed, we found that RORγt^K31R^, compared to WT RORγt and the controls RORγt^K11R^ and RORγt^K69R^, had impaired interactions with SRC1 (Fig. 7a). This finding was supported by the lower detection of endogenous RORγt^K31R^-SRC1 complexes in *RORγt*^*K31R/K31R*^ T_H_17 cells (Fig. 7b, left panel) and thymocytes (Fig. 7b, right panel) compared to their WT counterparts. Furthermore, our ChIP-seq assay showed that the recruitment of SRC1 to the *Il17a* and *Il17f* loci by RORγt^K31R^ was much less than that by WT RORγt in T_H_17 cells (Fig. 7c), again confirming reduced RORγt^K31R^-SRC1 interactions compared to RORγt-SRC1 interactions.

We found in the literature a report that KAT2A (or GCN5), a histone acetyltransferase, is able to synergize with SRC1 to bind to nuclear receptors^40^. Furthermore, using mass spectrometry, we identified with high confidence that KAT2A and SRC1 are RORγt-binding proteins in both thymocytes and T_H_17 cells (Supplementary Fig. 7b). Indeed, in HEK293T cells, we detected the RORγt–KAT2A interaction, which was further enhanced by the addition of SUMO3 and Ubc9. In contrast, the RORγt^K31R^–KAT2A interaction was much weaker and was not affected by SUMO3 and Ubc9 (Fig. 7d). These results suggest that the sumoylation of K31 promotes the interaction between RORγt and KAT2A. Furthermore, we found that the expression of KAT2A enhanced the RORγt–SRC1 interaction in HEK293T cells (Fig. 7e). On the other hand, knockdown of endogenous KAT2A greatly impaired the RORγt-SRC1 interaction in T_H_17 cells (Fig. 7f), suggesting that KAT2A promotes the RORγt–SRC1 interaction. In addition, immunoprecipitation of RORγt^K31R^ brought down much less KAT2A and SRC1 compared to immunoprecipitation of WT RORγt in both T_H_17 cells (Fig. 7b, left panel) and thymocytes (Fig. 7b, right panel), suggesting an essential role of K31 sumoylation in the formation of stable RORγt–KAT2A–SRC1 complexes. RORγt and SRC1 have already been established as essential for T_H_17 differentiation^13,25^, we thus aimed to determine the function of KAT2A in T_H_17 differentiation using a knockdown approach. We found that the knockdown of KAT2A (Supplementary Fig. 7c) impaired T_H_17 differentiation (Fig. 7g) and decreased expression of critical T_H_17 genes (Supplementary Fig. 7d). Of the two short hairpin RNAs used into knockdown KAT2A (shKAT2A-1 and shKAT2A-2), shKAT2A-2 inhibited T_H_17 differentiation more potently, which correlated with its higher potency in repressing KAT2A expression (Supplementary Fig. 7c), demonstrating an essential role of KAT2A in T_H_17 differentiation. Taken together, these data show that K31 sumoylation promotes the recruitment of KAT2A and SRC1 to RORγt to drive T_H_17 differentiation.

### PIAS4 catalyzes the K31 sumoylation of RORγt

**Fig. 8:**
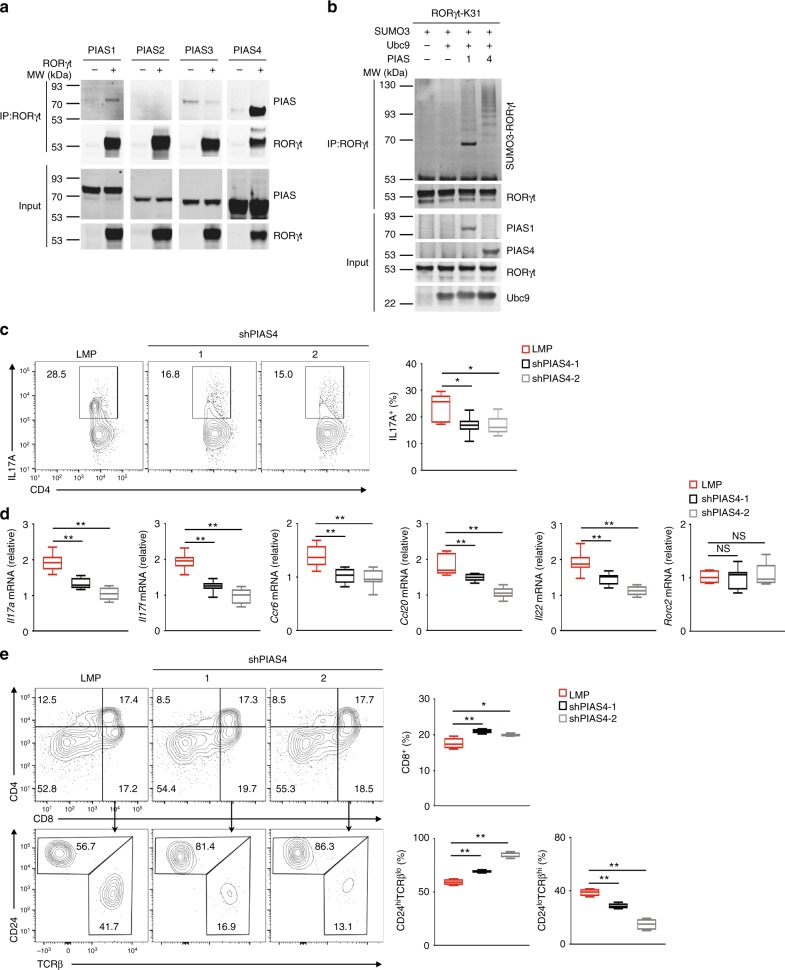
PIAS4 catalyzes the K31 sumoylation of RORγt and regulates RORγt-dependent functions. **a** Immunoblot analysis of different PIAS among immunoprecipitated RORγt from HEK293T cells expressing RORγt and various PIAS proteins. The bottom plots here and in **b** show immunoblot analysis of whole-cell lysates (input). **b** Immunoblot analysis of SUMO3-modified RORγt immunoprecipitated from HEK293T cells expressing various combinations of SUMO3, Ubc9, PIAS1 or PIAS4, and RORγt with all lysines except K31 mutated to arginines (RORγt-K31). **c** Cytometric analysis of the percentage of IL-17A^+^ cells (boxed) among WT CD4^+^ T cells transduced with retroviruses expressing GFP alone (LMP) or GFP with small hairpin RNA targeting PIAS4 (shPIAS4) and polarized for 3 d under T_H_17-priming conditions. The panel on the right presents the percentages of IL-17A^+^ cells among CD4^+^ cells from independent samples. **d** qPCR analysis of indicated mRNA in the T_H_17 cells assessed in **c**. Expression is presented relative to that of the control gene *Actb*. **e** flow cytometric analysis of CD4 and CD8 cells differentiated from CD4^−^CD8^−^thymocytes transduced with the retroviruses described in **c** and co-cultured for 3 d in vitro with OP9-DL4 stroma cells and IL-7 (5 ng/ml) to assess ex vivo thymocyte development (three top panels on the left). The top panel on the right presents the percentage of CD8^+^ thymocytes differentiated from independent samples. The bottom three panels on the left present the flow cytometry analysis of CD24 and TCRβ expression in CD8^+^ cells from the in vitro differentiated cells assessed in the top panels. The bottom two panels on the right present the percentages of immature TCR^lo^CD24^hi^ ISPs and mature TCR^hi^CD24^lo^ CD8^+^ cells differentiated in independent samples. NS, not significant (*P* > 0.05); *P < 0.05 (*t-*test); **P < 0.01 (*t-*test). Data are from three experiments (**c**, right; **d**; **e**, right; presented as median [central line], maximum and minimum [box ends], and outliers [extended lines]) or are one representative of three independent experiments (**a**; **b**; **c**, left panels**; e**, left panels)

PIAS proteins form the largest family of sumoylating E3 ligases^30^. To identify the E3 responsible for RORγt sumoylation, we first monitored the interactions between RORγt and individual PIAS proteins (Fig. 8a). We could not detect interactions between RORγt and PIAS2 or PIAS3. However, we detected a weak PIAS1–RORγt interaction and a strong PIAS4-RORγt interaction. We next sought to determine whether PIAS1 and PIAS4 could sumoylate RORγt at K31. For this purpose, we mutated all lysines of RORγt except K31 to arginines (RORγt-K31) so that only K31 could be sumoylated. Whereas we detected a relatively low amount of SUMO3-modified RORγt-K31 in the presence of PIAS1, we detected a much stronger SUMO3-modified RORγt-K31 signal in the presence of PIAS4 (Fig. 8b). As expected, we could barely detected any SUMO1-modified RORγt-K31 in the presence of PIAS1 or PIAS4 (Supplementary Fig. 8a), suggesting that PIAS4 and to a lesser extent PIAS1 can catalyze the addition of SUMO3, but not SUMO1, to K31 of RORγt.

To further evaluate the role of PIAS4 in regulating RORγt-dependent functions, we assessed the effects of PIAS4 knockdown on T_H_17 differentiation and thymocyte development in vitro. Knockdown of PIAS4 (Supplementary Fig. 8b) resulted in impaired T_H_17 differentiation (Fig. 8c) and reduced expression of important T_H_17 signature genes (Fig. 8d). However, knockdown of PIAS1 (Supplementary Fig. 8b) did not affect T_H_17 differentiation, which is consistent with a report that PIAS1 is not required for T_H_17 differentiation^41^. We next monitored the in vitro differentiation of sorted CD4^-^CD8^−^ thymocytes when PIAS1 or PIAS4 was knocked down. Knockdown of PIAS4 led to a slightly increased percentage of CD8^+^ SP cells (Fig. 8e, top panels). More importantly, there was a dramatically greater percentage of TCR^lo^CD24^hi^ CD8^+^ ISPs, and a correspondingly lower percentage of TCRβ^lo^CD24^hi^ CD8^+^ cells, among CD8^+^ SP cells in the cells with PIAS4 knocked down (Fig. 8e, bottom panels). In contrast, the knockdown of PIAS1 had no effect on the percentage of TCRβ^lo^CD24^hi^ CD8^+^ ISPs (Supplementary Fig. 8d). Therefore, the knockdown of PIAS4 impaired T_H_17 differentiation and increased ISPs, which were both phenotypes observed in *Sumo3*^*−/−*^ and *RORγt*^*K31R/K31R*^ mice. Taken together, these data suggest that PIAS4 catalyzes the addition of SUMO3 to K31 of RORγt and thus regulates RORγt-dependent T_H_17 differentiation and progression of ISPs.

## Discussion

RORγt controls the function of T_H_17 cells, which mediate both protective and pathogenic immunity. However, little is known about the post-translational mechanisms that regulate RORγt function. Our in vitro and in vivo results demonstrate that sumoylation of RORγt is a novel regulatory mechanism for controlling RORγt-dependent T_H_17 immunity, thymic ISP progression, and development of Peyer’s patches: (1) *Sumo3*^*−/−*^ but not *Sumo1*^*−/−*^ mice display defects in RORγt-regulated T_H_17 differentiation and thymocyte development (specifically, they accumulate ISPs); (2) RORγt is SUMO3- but not SUMO1-modified at K31 in both T_H_17 cells and thymocytes; (3) mice expressing RORγt^K31R^ exhibit multiple defective RORγt-dependent functions, including differentiation of T_H_17 cells, induction of T_H_17-mediated EAE, progression of ISPs in the thymus, and development of Peyer’s patches. We also identified PIAS4 as the E3 that catalyzes K31 sumoylation and regulates T_H_17 differentiation and progression of thymic ISPs. Therefore, we have demonstrated that post-translational sumoylation is a novel mechanism for modulating RORγt-dependent T_H_17 immunity that can be targeted by clinical therapies to enhance protective and inhibit pathogenic T_H_17 immunity.

Previous studies have reported that RORγt function is regulated by ubiquitination, which is a post-translational modification similar to but distinct from sumoylation^42,43^. The ubiquitin E3 ligase, Itch, was found to bind and ubiquitinate RORγt for degradation and thus regulate T_H_17-dependent immune responses^43^, which explains why *Itch*^*−/−*^ mice develop colitis. Another E3 ligase, UBR5, was also reported to regulate RORγt stability through the ubiquitin pathway^42^. However, the RORγt ubiquitination sites involved in the above two studies remain unknown. Meanwhile, we identified lysines 446 and 69 as ubiquitination sites through which RORγt-dependent T_H_17 differentiation can be controlled via degradation-independent mechanisms^33,44^. Therefore, we and others have demonstrated that T_H_17 immunity can be controlled through the ubiquitin pathway, which regulates RORγt stability and protein interactions. Although sumoylation can also regulate protein stability, our results do not support that K31 sumoylation affects RORγt stability. We showed that K31 sumoylation stimulates the recruitment of histone acetyltransferase KAT2A and co-activator SRC1 to RORγt. In addition, we showed that preventing K31 sumoylation reduces recruitment of SRC1 to the *Il17f* locus, suggesting that K31 sumoylation regulates the interaction between RORγt and its co-factors to activate *Il17f* expression.

RORγt has long been known to regulate thymocyte development^18^. However, RORγt chromatin occupancy and target genes in thymocytes were not known, which limited understanding of the mechanisms responsible for RORγt-regulated thymocyte development. To address this need, we mapped genome-wide RORγt DNA-binding sites and identified RORγt target genes. Furthermore, we identified K31 as the sumoylation site of RORγt, which enabled us for the first time to dissect RORγt functions in thymus. One important function of RORγt is to regulate thymocyte survival by up-regulating anti-apoptotic Bcl-x_L_ expression^18^. Our results showed that K31 sumoylation is actually not required to up-regulate Bcl-x_L_ or to maintain thymocyte survival; however, it is specifically required for the progression of thymic ISPs. Our study thus separates RORγt functions and establishes a link between RORγt-regulated functions and RORγt target genes.

T_H_17 cells produce the effector cytokines IL-17A, IL-17F, IL-22, and GM-CSF to mediate pathological inflammation responsible for many types of autoimmune diseases; targeting T_H_17 cells is thus a potential treatment for these diseases^45^. Indeed, inhibiting the T_H_17 pathway is effective for treating psoriasis and multiple sclerosis^46,47^. Considering the essential function of RORγt in T_H_17 cells, pharmaceutical and academic scientists are developing RORγt inhibitors to treat T_H_17-dependent autoimmunity^11,19,20,48,49^. Unfortunately, such RORγt inhibitors can induce thymic lymphoma by inhibiting RORγt during thymocyte development^50^. Although K31 sumoylation is required for the progression of thymic ISP, it is not essential for regulating thymocyte survival or cell cycle progression, which are most likely responsible for the development of lymphoma observed in *RORγt*^*−/−*^ mice^50,51^. Therefore, we expect that drugs targeting the K31 sumoylation pathway will inhibit T_H_17-mediated pathological immunity without interfering with thymocyte survival or cell cycle regulation, which could induce lymphoma in patients. Therefore, in addition to revealing a novel post-translational modification-based mechanism for regulating RORγt-dependent T cell function, our results also facilitate the development of a new category of RORγt-based drugs to treat T_H_17-mediated autoimmunity without serious side effects.

## Methods

### Mice

Both the targeting vector and the knock-in *RORγt*^*K31R/K31R*^ mice were designed and generated by Biocytogen LLC. *RORγt*^*K31R/K31R*^ mice are available at The Jackson Laboratory as Stock No. 032604. *Rag1*^*−/−*^ (002216) mice were purchased from the Jackson Laboratory. The *Rorγt*^*−/−*^ (*RORC2*^*−/−*^)^18^, *Sumo1*^*−/−*^^52^, and *Sumo3*^*−/−*^ mice^31^ were bred and housed under specific pathogen-free (SPF) conditions in the Animal Resource Center at the Beckman Research Institute of City of Hope under protocols approved by the Institutional Animal Care and Use Committee. Mice were 10–12 weeks of age for EAE studies and 6–8 weeks of age for all other experiments, with littermates age-matched across experimental groups.

### Antibodies and cytokines

Antibodies against RORγt (Q31-378, BD Bioscience, dilution ratio 1:1000), SRC1 (128E7, Cell Signaling, dilution ratio 1:1000), β-actin (SC-8422, Santa Cruz Biotechnology, dilution ratio 1:1000), GFP (A11122, Life technology, dilution ratio 1:1000), KAT2A (ab18381, Abcam, dilution ratio 1:1000), HA (HA-7, Sigma-Aldrich, dilution ratio 1:1000), FLAG (M2, Sigma-Aldrich, dilution ratio 1:5000), SUMO1 (C9H1, Cell Signaling Tech, dilution ratio 1:1000), SUMO3 (ab34661, Abcam, dilution ratio 1:1000), and PIAS4 (AV33011, Sigma-Aldrich, dilution ratio 1:1000) were used for immunoblot analysis. Phycoerythrin (PE)-indotricarbocyanine (Cy7)-conjugated anti-CD8 (53-6.7, dilution ratio 1:200), PE-conjugated anti-RORγt (B2D, dilution ratio 1:100), allophycocyanin (APC)-conjugated anti-IL-17A (eBio17B7, dilution ratio 1:100), PE-conjugated anti-Thy1.2 (53-2.1, dilution ratio 1:200), PE-conjugated anti-CD24 (M1/69, dilution ratio 1:100), PE-conjugated anti-TCRβ (H57-597, dilution ratio 1:100), PE-indodicarbocyanine (Cy5)-conjugated anti-CD19 (eBio1D3, dilution ratio 1:100), PE-conjugated anti-CD11b (M1/70, dilution ratio 1:100), fluorescein isothiocyanate (FITC)-conjugated anti-CD4 (GK1.5, dilution ratio 1:200), APC-conjugated anti-IL-4 (11B11, dilution ratio 1:100), and APC-conjugated anti-Foxp3 (FJK-16s, dilution ratio 1:100) antibodies were from eBioscience. Monoclonal antibodies against mouse CD3 (145-2C11), CD28 (37.51), IL-4 (11B11), IFN-γ (XMG1.2), and the p40 subunits of IL-12 and IL23 (C17.8), as well as PE-Cy7-conjugated anti-Ly6G (1A8, dilution ratio 1:100), FITC-conjugated anti-IFN-γ (XMG1.2, dilution ratio 1:100), PE-conjugated anti-GM-CSF (MP1-22E9, dilution ratio 1:100), FITC-Cy7-conjugated anti-CD45 (104, dilution ratio 1:200), and PE-conjugated anti-CD25 (PC61.5, dilution ratio 1:100) antibodies, were purchased from BioLegend. Goat anti-hamster antibody was from MP Biomedicals. APC-conjugated anti-CD3 (UCHT1, dilution ratio 1:200) and FITC-conjugated anti-CD44 (IM7, dilution ratio 1:100) antibodies were from BD Pharmingen. Recombinant mouse IL-12, IL-4, IL-6, IL-23, and TGFβ were from Miltenyi Biotech. Recombinant mouse IL-2 was from Pepro Tech. The antibody against RORγt used for ChIP was a generous gift from Dan Littman at New York University.

### Plasmids

cDNA encoding RORγt or SRC1 was inserted into a XhoI/EcoRI-cut pMSCV vector^44^. Point mutations of RORγt were generated using a site-directed mutagenesis kit from Agilent Technologies. pRK5-HA-ubiquitin (a gift from Ted Dawson at Johns Hopkins University School of Medicine; #17603-17608), pCMV-sport2-mGCN5 (a gift from Sharon Dent at MD Anderson Cancer Center; #23098), and constructs for expressing FLAG-PIAS (gifts from Ke Shuai at the University of California Los Angeles; #15206-15210) were obtained from Addgene. pCMV-FLAG-SUMO1, pCMV-FLAG-SUMO3, and pcDNA-UBC9 were generous gifts from Yuan Chen at the City of Hope. The LMP vector-based retroviral short hairpin RNA (shRNA)-expressing vectors were constructed using following oligonucleotide sequences: shKAT2A-1: TGCTGTTGACAGTGAGCGACCGCTATCTGGGCTACATCAATAGTGAAGCCACAGATGTATTGATGTAGCCCAGATAGCGGCTGCCTACTGCCTCGGA; shKAT2A-2: TGCTGTTGACAGTGAGCGCGCCAAGAATGCCCAAGGAATATAGTGAAGCCACAGATGTATATTCCTTGGGCATTCTTGGCATGCCTACTGCCTCGGA; shPIAS1-1: TGCTGTTGACAGTGAGCGAGGAACTAAAGCAAATGGTTATTAGTGAAGCCACAGATGTAATAACCATTTGCTTTAGTTCCGTGCCTACTGCCTCGGA; shPIAS1-2: TGCTGTTGACAGTGAGCGCCCGGATCATTCTAGAGCTTTATAGTGAAGCCACAGATGTATAAAGCTCTAGAATGATCCGGATGCCTACTGCCTCGGA; shPIAS4^−^1: TGCTGTTGACAGTGAGCGCGCTACAGAGGTTGAAGACGATTAGTGAAGCCACAGATGTAATCGTCTTCAACCTCTGTAGCATGCCTACTGCCTCGGA; shPIAS4-2: TGCTGTTGACAGTGAGCGCGAGCTGTATGAGACTCGCTATTAGTGAAGCCACAGATGTAATAGCGAGTCTCATACAGCTCTTGCCTACTGCCTCGGA.

### Retrovirus transduction

Platinum-E packaging cells (Cell Biolabs) were plated in a 10-cm dish in 10 ml RPMI-1640 medium plus 10% FBS. 24 h later, cells were transfected with empty pMSCV or pLMP vectors or the appropriate retroviral expression plasmids with BioT transfection reagent (Bioland). After overnight incubation, the medium was replaced and cultures were maintained for another 24 h. Viral supernatants were collected 48 and 72 h later, passed through 0.4-μm filters (Millipore), and supplemented with 8 µg/ml of polybrene (Sigma-Aldrich) and 100 U/ml of recombinant IL-2 (for transducing CD4^+^ T cells) or 5 ng/ml of recombinant IL-7 (for transducing CD4^-^CD8^−^ thymocytes). Naïve CD4^+^ T cells were first activated with 0.25 µg/ml hamster anti-CD3 (145-2C11; Biolegend) and 1 µg/ml hamster anti-CD28 (37.51; Biolegend) in 24^−^well plates pre-coated with 0.2 mg/ml goat anti-hamster antibody for 24 h, then spin-infected with viral supernatants (1200 g, 30°C for 2 h). The retroviral supernatant was also used to infect CD4^−^CD8^−^ thymocytes that had been co-cultured with feeder OP9-DL4 cells (a generous gift from Ellen Rothenberg at Caltech) in the presence of recombinant IL-7 (5 ng/ml) for 24 h. After spin infection, the viral supernatant was replaced with culture media containing polarizing cytokines for in vitro differentiation (for transduced CD^+^ T cells) or 5 ng/ml of recombinant IL-7 for in vitro T cell development (for transduced CD4^−^CD8^−^ thymocytes), as described below.

### In vitro differentiation

Naïve CD4^+^ T cells were purified from C57BL/6, *RORγt*^*−/−*^, or *RORγt*^*K31R/K31R*^ mice by negative selection (Miltenyi Biotec). Suspensions of 4 × 10^5^ cells/ml Iscove’s modified DMEM (Cellgro) containing 2 mM L-glutamine, 50 mM 2-ME, 100 U/ml penicillin, 100 mg/ml streptomycin, and 10% FBS were cultured in 24-well plates pre-coated with 0.2 mg/ml goat anti-hamster antibody for three days. The medium was supplemented with 0.25 µg/ml hamster anti-CD3, 1 µg/ml hamster anti-CD28, and polarizing cytokines: 2 ng/ml TGF-β, 20 ng/ml IL-6, 5 µg/ml anti-IL-4, and 5 µg/ml anti-IFNγ for T_H_17 differentiation; 20 µg/ml IL-12 and 5 µg/ml anti-IL-4 for Th1 differentiation; 10 ng/ml IL-4 and 10 µg/ml anti-IFNγ for T_H_2 differentiation; or 5 ng/ml TGF-β for Treg differentiation. For analysis, cells obtained from in vitro cultures were incubated for 4–5 h with 50 ng/ml PMA (Sigma-Aldrich), 750 ng/ml ionomycin (Sigma-Aldrich), and 10 µg/ml brefeldin A (BD Biosciences) in a tissue culture incubator at 37 °C, followed by intracellular cytokine staining.

### In vitro T cell development

Thymocytes were stained with 7-AAD and antibodies against Thy1.2, CD4, and CD8. Specific 7-AAD^−^Thy1.2^+^CD4^−^CD8^−^populations were sorted using a FACSAria (BD Biosciences) and cultured at 5 × 10^5^/ml overnight on an 80% confluent OP9-DL4 monolayer in flat-bottom 24-well culture plates with αMEM (MEM α medium; Invitrogen Life Technologies) supplemented with 20% FBS, 100 U/ml penicillin–streptomycin, 2 mM L-glutamine (Invitrogen Life Technologies), and 5 ng/ml recombinant murine IL-7. After 72 h, co-cultures were harvested for flow cytometry analysis.

### Flow cytometry

Mouse thymi or spleens were homogenized by crushing with the head of a 1-ml syringe in a petri dish, followed by straining through a 40-μm nylon filter. Red Blood Cell Lysing buffer (Sigma-Aldrich) was used for red cell lysis. Cells isolated from thymi or spleens, co-cultures harvested from in vitro development, and CD4^+^ T cells stimulated appropriately were stained for surface markers. Intracellular cytokines were stained with Fixation/Permeabilization solution (BD Cytofix/Cytoperm Kit; BD Biosciences). The expression of surface and intracellular markers were analyzed with FACSCanto (BD).

### RNA sequencing and analysis

To measure gene expression in the thymi of WT or *RORγt*^*K31R/K31R*^ mice, two separate samples were collected on different days, and thymocytes from four (two male and two female) were pooled each day. To determine the gene expression profile of T_H_17 cells, naive CD4^+^ T cells were enriched from WT or *RORγt* ^*K31R/K31R*^ mice and polarized under T_H_17 conditions for three days. Cells were processed for RNA isolation (Qiagen). Quality verification, library preparation, and sequencing were performed at the City of Hope Integrative Genomics Core Facility. Eluted RNAs were prepared for sequencing using Illumina protocols and sequenced on an Illumina HiSeq 2500 to generate 51-bp reads. Sequenced reads were aligned to the mouse mm10 reference genome using TopHat. Gene expression levels were quantified using HTSeq, and edgeR was utilized to identify differentially expressed genes (fold-change > 1.5 and FDR < 0.05). Gene expression abundance was quantified as fragments per kilobase of transcript per million fragments mapped (FPKM). Heat maps of differentially expressed genes were made with gplots using log2-transformed FPKM values.

### Chromatin immunoprecipitation and DNA sequencing (ChIP-seq)

A total of 2 × 10^7^ cells were incubated with 1% formaldehyde to cross-link proteins with chromatin for 5 min at room temperature. 125 mM glycine was added to stop the cross-linking reaction. To shear genomic DNA into 200–500-bp fragments, cell lysates were sonicated using a water-bath sonicator (Covaris S200). Cell lysates were centrifuged (12,000 ×  *g*, 10 min) and incubated with specific antibodies (anti-RORγt from D. Littman or anti-SRC1 from Abcam) or IgG controls and protein A/G beads (Millipore). After extensive washing, DNA was eluted followed by reversion of the protein–DNA cross-linking. DNA was recovered for sequencing or qRT-PCR to quantify specific DNA fragments that were precipitated. Primers used for qRT-PCR are listed in Supplementary Table 1. Two biological replicates for each condition were sequenced on an Illumina HiSeq 2500 to produce 51-bp reads. Reads were aligned to the mm10 mouse genome using NovoAlign (http://www.novocraft.com/). TDF files were generated for visualization on the Integrative Genomics Viewer^53^. The enrichment of RORγt binding sites across the genome was analyzed using MACS2 with ‘—nomodel—extsize 150’^54^. The irreproducible discovery rate (IDR) framework was utilized find reproducible peaks across replicates. Enriched known TF motifs in ChIP-seq peaks were identified by using HOMER (findMotifsGenome.pl)^55^.

### Quantitative real-time PCR

qRT-PCR was performed using SsoFast EvaGreen Supermix (Bio-Rad) in a CFX96 Real-Time PCR Detection System (Bio-Rad), using the primers listed in Supplementary Table 1. The amplification efficiency of all primers was previously tested, and the optimized conditions were used for all qRT-PCR reactions. Expression was calculated using the ΔΔxp method normalized to *β-actin*, and all measurements were performed in triplicate.

### Apoptosis assays

Thymocytes were freshly isolated from WT, *RORγt*^*−/−*^, or *RORγt*^*K31R/K31R*^ mice and cultured in RPMI 1640 medium supplemented with 10% FBS, 100 U/ml penicillin–streptomycin, and 2 mM L-glutamine at 1 × 10^6^ cells/ml. Thymocytes were incubated at 37 °C with 5% CO_2_. Dead cells were detected using Annexin V-PE and 7-AAD staining (BD Bioscience).

### Induction and assessment of EAE

Active EAE was induced using an EAE induction kit, according to the manufacturer’s instructions (Hooke Laboratories, Lawrence, MA). Briefly, mice were subcutaneously immunized with a 200-ml myelin oligodendrocyte glycoprotein 35–55 (MOG_35–55_) peptide emulsion. On days 0 and 1 after immunization, mice were injected intraperitoneally with 200 ng *Bordetella pertussis* toxin. For T_H_17- or T_H_1-induced passive EAE, donor mice were immunized with MOG_35–55_ subcutaneously. 10 days later, cells were isolated from the spleen and lymph nodes and cultured with 20 µg/ml MOG_35–55_ for 3 days under either T_H_17-polarizing conditions (20 ng/ml rmIL23) or T_H_1-polarizing conditions (20 ng/ml rmIL-12; 2 µg/ml α-IL23p19). *Rag1*^*−/−*^ recipient mice were then intraperitoneally transferred 3.0 × 10^7^ MOG_35–55_-specific T_H_17 or T_H_1 cells. The severity of EAE was monitored and evaluated on a scale from 0 to 5 according to the Hooke Laboratories guidelines: 0 = no disease; 1 = paralyzed tail; 2 = hind limb weakness; 3 = hind limb paralysis; 4 = hind and forelimb paralysis; and 5 = moribund and death. When a mouse was euthanized because of severe paralysis, a score of 5 was entered for that mouse for the rest of the experiment.

### Immunoprecipitation and immunoblot analysis

Cells were lysed in lysis buffer (1% Triton X-100, 20 mM Tris-cl, pH 7.4, 150 mM NaCl, and 5 mM EDTA) supplemented with protease inhibitor cocktail (Sigma) and 1 mM PMSF. Cell extracts were incubated overnight with 1 µg of the relevant antibodies, and proteins were immunoprecipitated for an additional 1 h at 4 °C with protein A/G-Sepharose beads (milipore). To detect sumoylation, transfected HEK293T cells, primary thymocytes, or polarized T_H_17 cells were lysed in lysis buffer containing 20 mM N-ethylmaleimide. Supernatant was supplemented with 1% SDS (vol/vol) and heated at 90 °C for 10 min. Samples were then diluted (1:10) with lysis buffer and incubated with anti-RORγt at 4 °C overnight. Enrichment of ubiquitinated proteins was performed as previously described^44^. Briefly, cell lysates were incubated with equilibrated Agarose-coupled Tandem Ubiquitin Binding Entity 1 (Agarose-TUBE1) (LifeSensors) at 4 °C for 4 h. After incubation, beads were washed four times with lysis buffer, resolved using SDS-PAGE, and analyzed using Western blot.

### Statistical analysis

Prism software (GraphPad) was used for all statistical analyses. Two-tailed unpaired Student’s *t*-tests and one-way analysis of variance (ANOVA) were used to compare experimental groups. A *P*-value of less than 0.05 was considered statistically significant.

## Electronic supplementary material

Supplementary Information

Peer Review file

## Data Availability

The data that support the findings of this study are available from the corresponding author upon request. The SRA (Sequence Read Archive) accession code for RNA-seq and ChIP-seq data is SRP150962.
