# Supplementary material for: Sumoylation of RORγt regulates TH17 differentiation and thymocyte development
Source: Nat Commun. 2018 Nov 19;9:4870. doi: 10.1038/s41467-018-07203-z (PMC6242824; doi:10.1038/s41467-018-07203-z)
Supplement: Supplementary file 1 — Supplementary Information [file 41467_2018_7203_MOESM1_ESM.pdf]

Supplementary Information

Sumoylation of RORγt regulates T_H17 differentiation and thymocyte development

He et al.

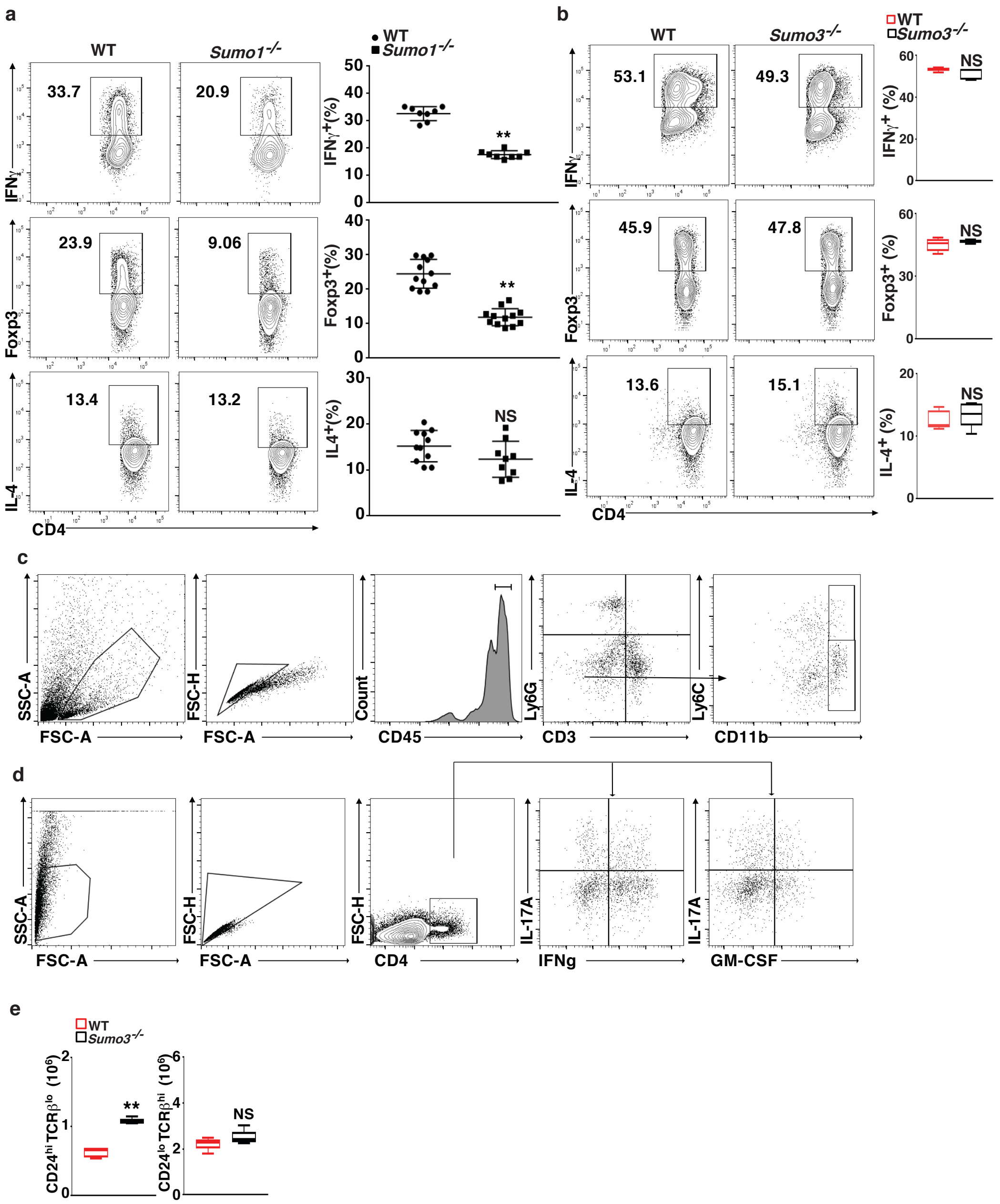

Supplementary Figure 1. SUMO3 but not SUMO1 facilitates T_H17 differentiation and ISP matureation. (**a & b**) Representative flow cytometric analysis of intracellular IFNγ (top), Foxp3 (middle), and IL-4 (bottom) cells among WT and (**a**) *Sumo1*$^{-/-}$ or (**b**) *Sumo3*$^{-/-}$ naïve CD4^{+} T cells differentiated for 3 d under T_H1, Treg, and T_H2 priming conditions, respectively. The percentages of the corresponding populations among CD4^{+} T cells from independent samples are shown on the right. (**c**) Gating strategy for **Fig. 1d**. (**d**) Gating strategy for **Fig. 1e**. (**e**) Absolute numbers of CD24^{hi}TCR^{lo} and CD24^{lo}TCR^{hi} thymocytes in the thymi of the individual mice assessed in **Fig. 2f** (*n* = *5* per genotype). NS, not significant (P>0.05); ** P <0.01 (*t-test*). Data are from three experiments (**a**, right; **e**; presented as [central line], maximum and minimum [box ends], and outliers [extended lines]; **b**, right; mean ± s.e.m) or are one representative of three independent experiments (**a** and **b**, left**;** **c** and **d**).

a

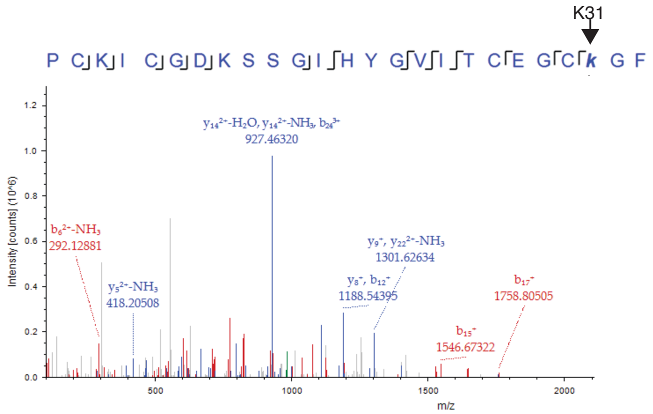

b

Species	Sequence around K31	Accession Number
Mus musculus	GIHYGVITCEGCKGFFRRSQQ	NM_001293734
Homo sapiens	GIHYGVITCEGCKGFFRRSQR	NM_001001523
Rattus norvegicus	GIHYGVITCEGCKGFFRRSQQ	XM_008761339
Danio rerio	GVHYGVITCEGCKGFFRRSQQ	XM_001344013
Pan troglodytes	GIHYGVITCEGCKGFFRRSQQ	XM_003308391
Gallus gallus	GIHYGVITCEGCKGFFRRSQQ	XM_025143431
Xenopus laevis	GIHYGVITCEGCKGFFRRSQQ	NM_001094920

c

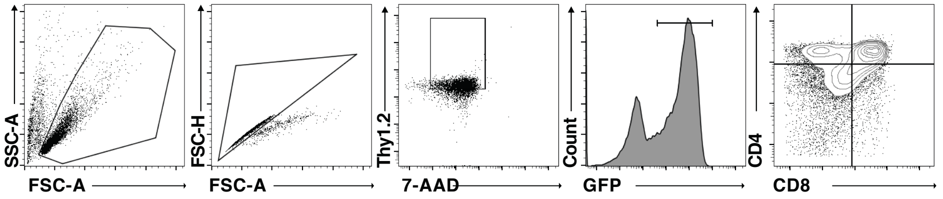

d

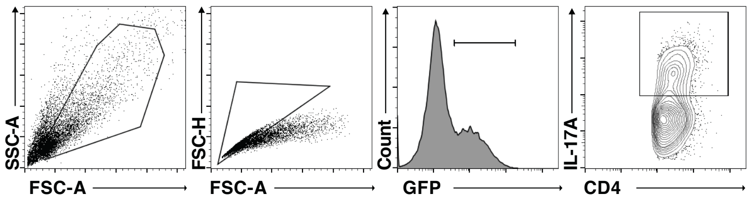

Supplementary Figure 2. Identification of K31 as a sumoylation site of RORγt. (**a**) Representative mass spectrometry analysis of RORγt immunoprecipitated (using an anti-RORγt antibody) from CD4$^+$ T cells obtained from WT mice and polarized *in vitro* for 3 d under T$_H$17 conditions. RORγt-K31 (indicated with an arrow) was identified as a residue conjugated with SUMO. (**b**) Analysis of homologous amino acids (marked in light gray) flanking K31 (marked in red) within RORγt homologs from various species. (**c**) Gating strategy for **Fig. 3d**. (d) Gating strategy for **Fig. 3g**.

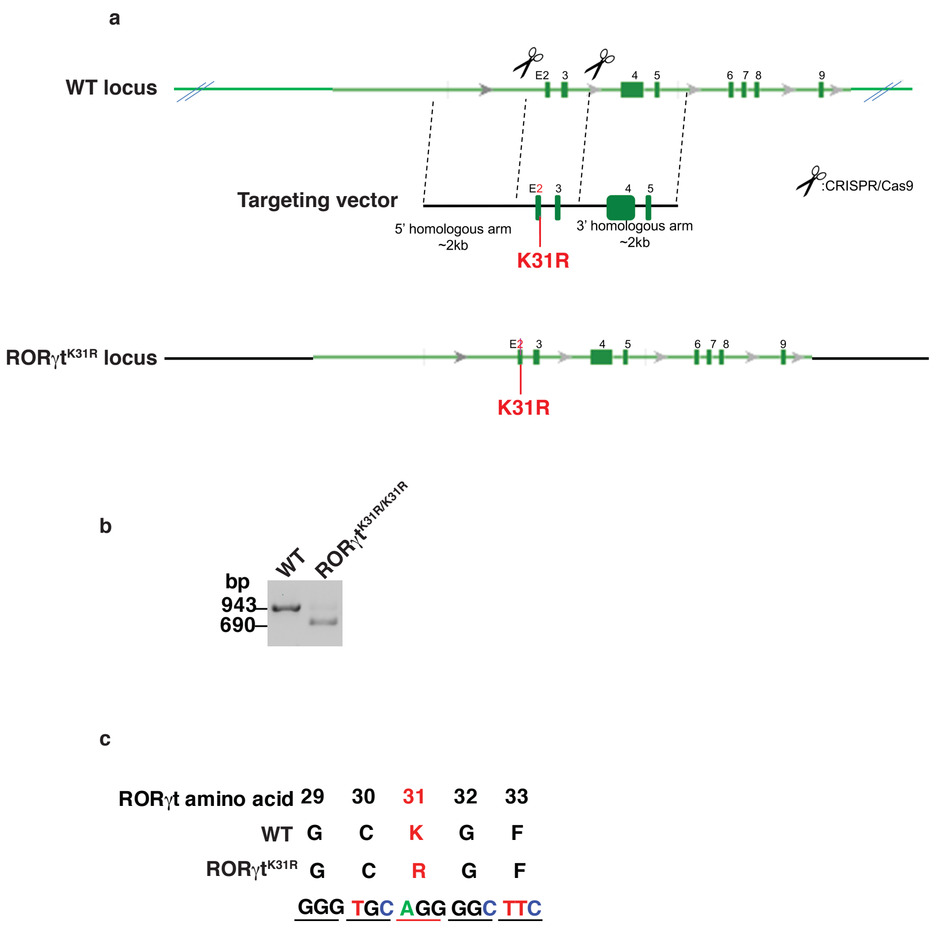

Supplementary Figure 3. Generation of RORγt$^{K31R/K31R}$ mice. (**a**) Strategy to generate RORγt$^{K31R/K31R}$ mice using the Crispr/Cas9 system. (**b**) Genotyping analysis of WT and RORγt$^{K31R/K31R}$ mice. The sizes of the bands, in base pairs (bp), are shown on the left. (**c**) DNA sequencing analysis confirming the genotype of WT and RORγt$^{K31R/K31R}$ mice.

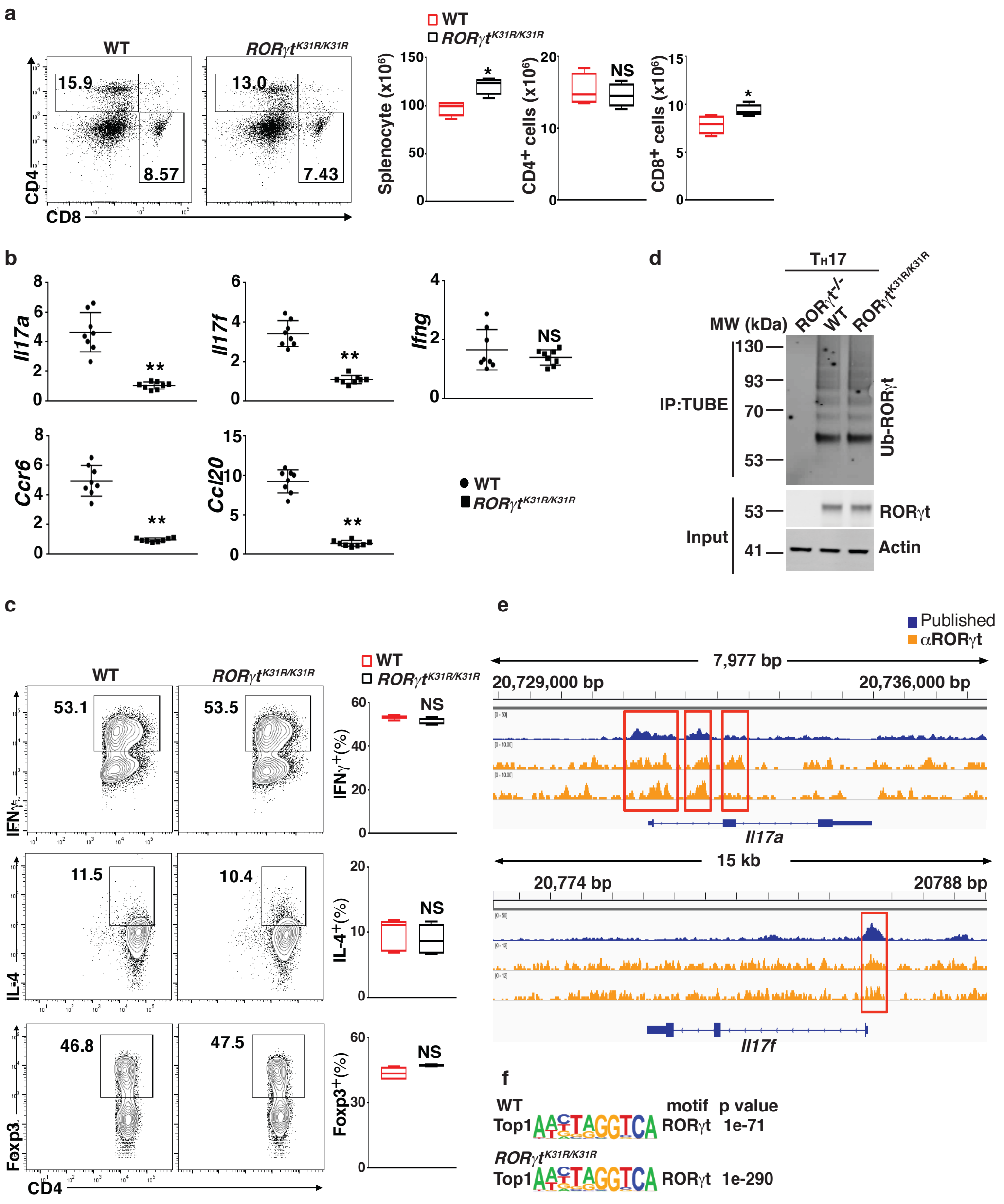

Supplementary Figure 4. CD4$^+$ T cells from RORγt$^{K31R/K31R}$ mice exhibit defective T$_H$17 differentiation. (**a**) Representative flow cytometric analysis of CD4 and CD8 on the surface of the splenocytes of WT and RORγt$^{K31R/K31R}$ mice. The total splenocyte numbers and absolute numbers of CD4$^+$ and CD8$^+$ lymphocytes in individual mice (*n = 4* per genotype) are shown on the right. (**b**) qPCR analysis of *Il17a* (top left), *Il17f* (top middle), *Ifng* (top right), *Ccr6* (bottom left), and *Ccl20* (bottom middle) mRNA in the T$_H$17 cells assessed in **Fig. 4a**. Expression is presented relative to that of the control gene *Actb*. (**c**) Representative flow cytometric analysis of intracellular IFNγ (top), IL-4 (middle), and Foxp3 (bottom) among naïve CD4$^+$ T cells in WT and *RORγt$^{K31R/K31R}$* mice differentiated for 3 d under T$_H$1, Treg, T$_H$2 priming conditions, respectively. The frequencies of the corresponding populations among CD4$^+$ T cells from individual mice

(*n = 4* per genotype) are shown on the right. (**d**) Immunoblot analysis of RORγt among ubiquitinated proteins enriched with TUBE-1 agarose from CD4$^+$ cells of *RORγt*$^{-/-}$, WT or *RORγt*$^{K31R/K31R}$ mice polarized under T_H17 conditions. (**e**) RORγt DNA-binding peaks in *Il17a* (top) and *Il17f* (bottom) identified by ChIP analysis using an anti-RORγt (orange) antibody compared with those idenfied using anti-FLAG (published; blue). (**f**) *De novo* motif enrichment analysis of RORγt footprints in the promoter regions identified by ChIP, as shown in **Fig. 4f**. NS, not significant (P>0.05); * P < 0.05 (*t-test*); ** P <0.01 (*t-test*). Data are from three experiments (**a**, right; **c**, right; presented as median [central line], maximum and minimum [box ends], and outliers [extended lines]; b; mean ± s.e. m), two biological replicates (**f**), or are one representative of three independent experiments (**a**, left; **c**, left; **d**; **e**).

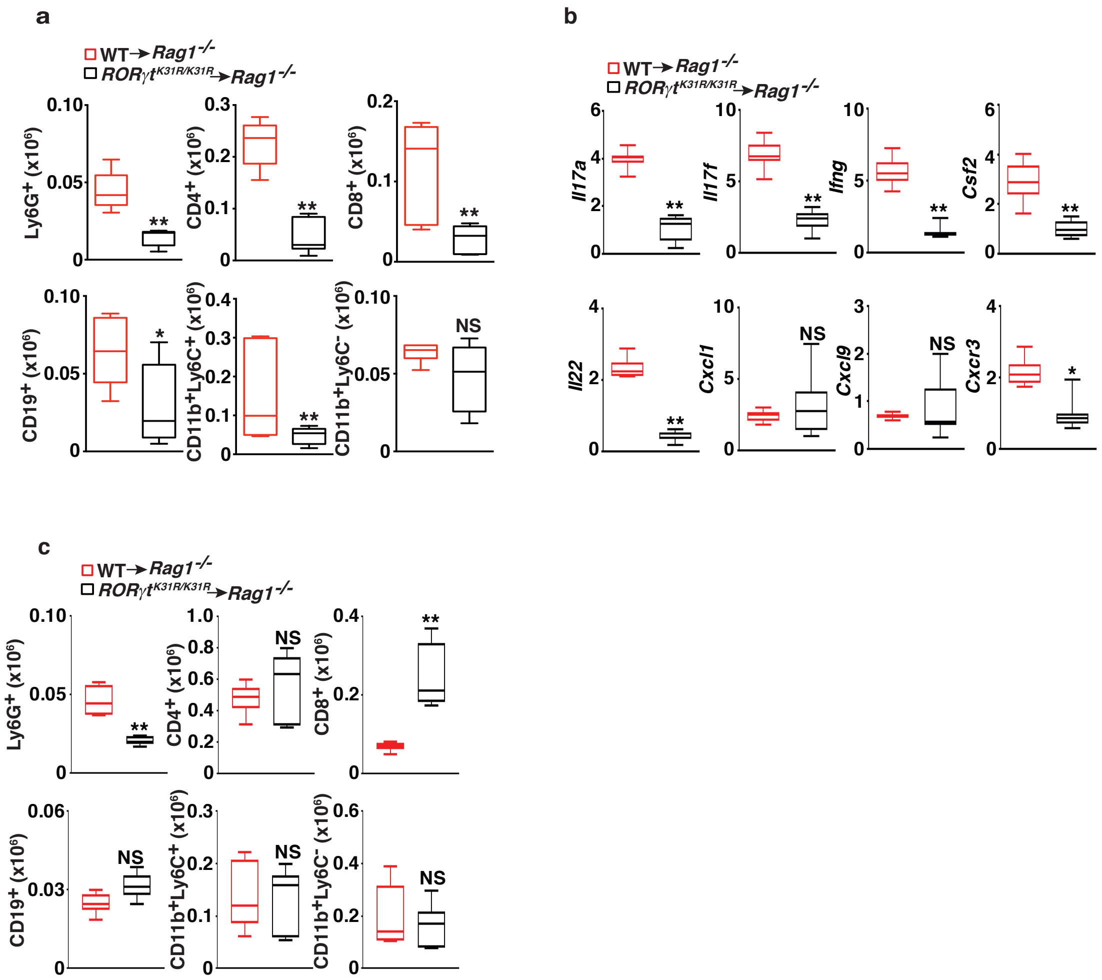

Supplementary Figure 5 RORγt-K31 sumoylation deficiency attenuates $\mathrm{T_H}$17-mediated but not $\mathrm{T_H}$1-mediated EAE. (**a**) Quantification of CNS-infiltrating cells from WT and $ROR\gamma t^{K31R/K31R}$ mice (*n* = *5* per genotype, assessed in **Fig. 5d**) expressing characteristic mononuclear cell surface markers, assessed using flow cytometry at the peak of disease. (**b**) qPCR analysis of cytokine-encoding *Il17a* (top left), *Il17f* (top middle), *Ifng* (top right), *Csf2* (bottom left), *Il22* (bottom middle), and *Ccl20* (bottom right) mRNA in lymphocytes that infiltrated the CNS of mice (*n* = *5* per genotype, assessed in **Fig. 5d**). Expression is presented relative to that of the control gene *Actb*. (**c**) Quantification of CNS-infiltrating cells from WT and $ROR\gamma t^{K31R/K31R}$ mice (*n* = *5* per genotype, assessed in **Fig. 5e**) expressing characteristic mononuclear cell surface markers, assessed using flow cytometry at the peak of disease. NS, not significant (P>0.05); * P < 0.05 (*t-test*); ** P <0.01 (*t-test*). Data are from three experiments (**a**–**c**; presented as median [central line], maximum and minimum [box ends], and outliers [extended lines]).

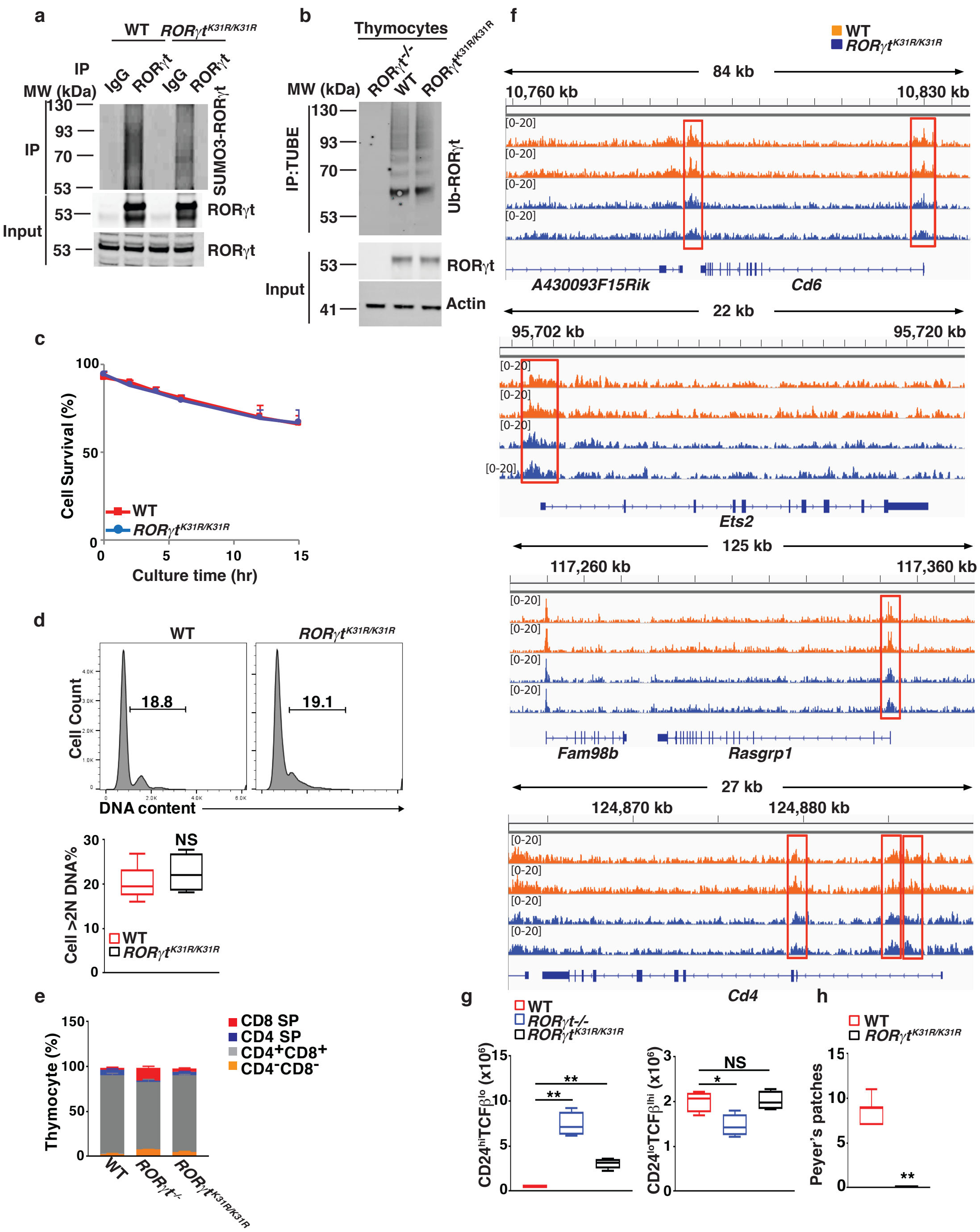

Supplementary Figure 6. RORγt$^{K31R/K31R}$ mice accumulate thymic ISPs and lack Peyer's patches. (**a**) Immunoblot analysis of SUMO3-modified RORγt immunoprecipitated using IgG or anti-RORγt antibodies from WT or *RORγt$^{K31R/K31R}$* thymocytes. The bottom panel shows the immunoblot analysis of total RORγt, used as a loading control (input). (**b**) Immunoblot analysis of RORγt among ubiquitinated proteins enriched with TUBE-1 agarose from WT, *RORγt$^{-/-}$*, or *RORγt$^{K31R/K3}$* thymocytes. The bottom panel shows the immunoblot analysis of total RORγt and Actin, used as loading controls. (**c**) Frequency of surviving cells among thymocytes obtained from WT and *RORγt$^{K31R/K31R}$* mice (*n* = 5 per

genotype), cultured for 1–15 h, stained for the apoptosis marker annexin V and with the membrane-impermeable DNA-interacting dye 7-AAD, and analyzed by flow cytometry. (**d**) Representative flow cytometric analysis of the DNA content (according to 7-AAD staining) of WT and *RORγt*$^{K31R/K31R}$ thymocytes. The bottom panel presents the frequencies of cells with >2N DNA from individual mice (*n* = *5* mice per genotype). Numbers above bracketed lines (top) indicated percent cells with >2N DNA. (**e**) The frequencies of $CD8^+$, $CD4^+$, $CD4^+CD8^+$, and $CD4^-CD8^-$ populations among thymocytes obtained from WT and *RORγt*$^{K31R/K31R}$ mice. (**f**) ChIP analysis identifying RORγt DNA-binding peaks in *Cd6*, *Ets2*, *Rasgrp1*, and *Cd4* in cells assessed in **Fig. 6i**. (**g**) Absolute numbers of $CD24^{hi}TCR^{lo}$ and $CD24^{lo}TCR^{hi}$ thymocytes from WT, *RORγt*$^{-/-}$, or *RORγt*$^{K31R/K3}$ mice (*n* = *5* per genotype, assessed in **Fig. 6d**). (**h**) Quantification of Peyer's patches obtained from WT and *RORγt*$^{K31R/K31R}$ mice (*n* = *5* per genotype). NS, not significant (P>0.05); ** P <0.01 (*t-test*). Data are from three experiments (**a**; **b**, bottom; **c**; **d**; **f**; presented as median ([central line], maximum and minimum [box ends], and outliers [extended lines]), two biological replicates (**e**), or are one representative of three independent experiments (**b**, top).

a

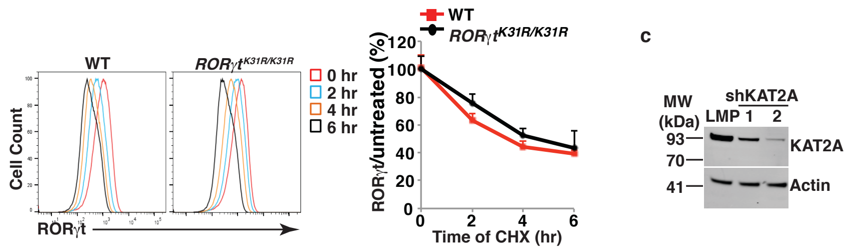

b

Accession	Description	Coverage	Unique Peptides	Peptides	Score	
84000003;Q3TZ9	histone acetyltransferase KAT2A isoform a [Mus musculus]	20.36	9	10	116.8585	T_H17 cells
6754800	nuclear receptor coactivator 1 [Mus musculus]	5.27	3	3	21.74142	

Accession	Description	Coverage	Unique Peptides	Peptides	Score	
84000003;Q3TZ59	histone acetyltransferase KAT2A isoform a [Mus musculus]	14.84	7	7	115.15	Thymocytes
6754800	nuclear receptor coactivator 1 [Mus musculus]	10.32	4	6	41.73	

d

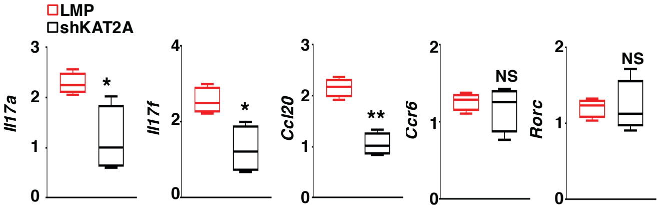

Supplementary Figure 7. Sumoylation of RORγt-K31 stimulates the recruitment of KAT2A and co-activator SRC1. (**a**) Representative flow cytometric analysis of RORγt in the cells assessed in **Fig. 4a** at serial time points after incubation with the protein synthesis inhibitor cycloheximide (CHX). The percentages of RORγt$^+$ cells among original T_H17 cells from WT and *RORγt*$^{K31R/K31R}$ mice ($n = 5$ per genotype) are shown on the right. (**b**) Mass spectrometry results identifying KAT2A and SRC1 among the proteins immunoprecipitated from CD4$^+$ T cells obtained from WT mice using an anti-RORγt antibody and polarized for 3 d *in vitro* under T_H17 conditions (top) or thymocytes from the same mice. (**c**) Immunoblot analysis of the knockdown efficiency of the KAT2A-targeting shRNAs used to generated data presented in **Fig. 7f & 7g**. (**d**) qPCR analysis of T_H17 signature genes: *Il17a* (left), *Il17f* (second from left), *Ccl20* (middle), *Ccr6* (second from right), and *Rorc* (right) mRNA in the cells assessed in **Fig. 7g**. Expression is presented relative to that of the control gene *Actb*. NS, not significant (P>0.05); ** P <0.01 (*t-test*). Data are from three experiments (**a**, right; presented as mean ± s.e.m; **d**; presented as median [central line], maximum and minimum [box ends], and outliers [extended lines]), or are one representative of three independent experiments (**a**, left; **c**).

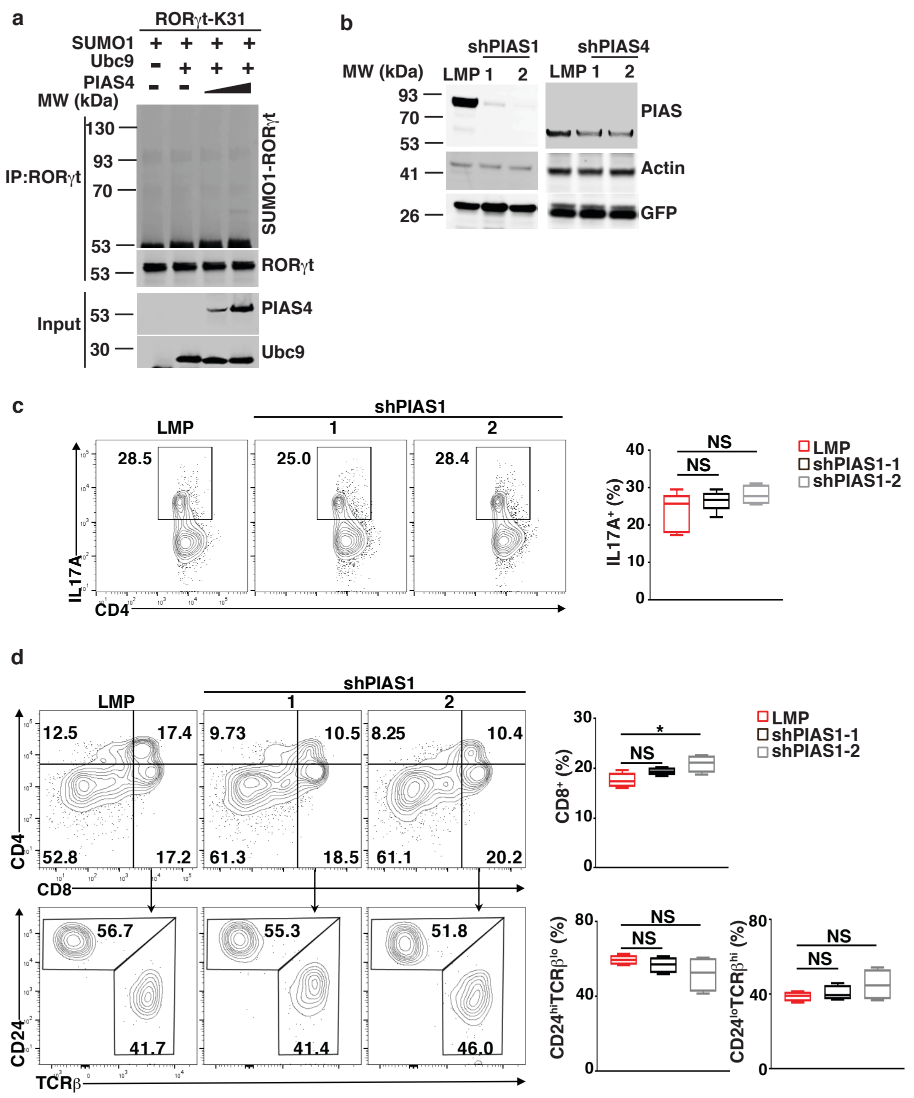

Supplementary Figure 8. PIAS4 catalyzes the sumoylation of RORγt-K31 and regulates RORγt-dependent functions. (**a**) Immunoblot analysis of SUMO1-sumoylated RORγt immunoprecipitated with an anti-RORγt antibody in HEK293T cells co-transfected with plasmids to express SUMO1, Ubc9, RORγt with all lysines except K31 mutated to argnines (RORγt-K31), and PIAS4. (**b**) Immunoblot analysis of the knockdown efficiency of the PIAS-targeting shRNAs used to generate the data presented in **Fig. 8c & 8e** and **Supplementary Fig. 8c & 8d**. (**c**) Representative flow cytometric analysis of IL-17A$^+$ cells (boxed) among WT CD4$^+$ T cells transduced with retroviruses expressing GFP alone (LMP) or GFP with shRNA targeting PIAS1 (shPIAS1) and polarized for 3 d under T$_H$17-priming conditions. The frequencies of IL-17A$^+$ cells among CD4$^+$ cells from independent samples are shown on the right. (**d**) Representative flow cytometric analysis of CD4 and CD8 expression by CD4$^-$CD8$^-$ thymocytes sorted from WT mice, transduced with the retroviruses described in **c** and cultured for 3 d *in vitro* in the presence of OP9-DL4 stroma cells and IL-7 (5 ng/ml) to assess *ex vivo* thymocyte development. The frequencies of CD8$^+$ thymocytes from independent samples are shown on the right. The panels on the bottom present flow cytometric analysis of CD24 and TCRβ expression in

the $CD8^+$ subpopulation from the top panels. The frequencies of $CD24^{hi}TCR^{lo}$ and $CD24^{lo}TCR^{hi}$ thymocytes from independent samples are shown on the bottom right. NS, not significant ($P>0.05$); * $P < 0.05$ (*t-test*). Data are from three experiments (**c** and **d**, right; presented as median [central line], maximum and minimum [box ends], and outliers [extended lines]) or are one representative of three independent experiments (**a**; **b**; **c**, left**;** **d**, left).

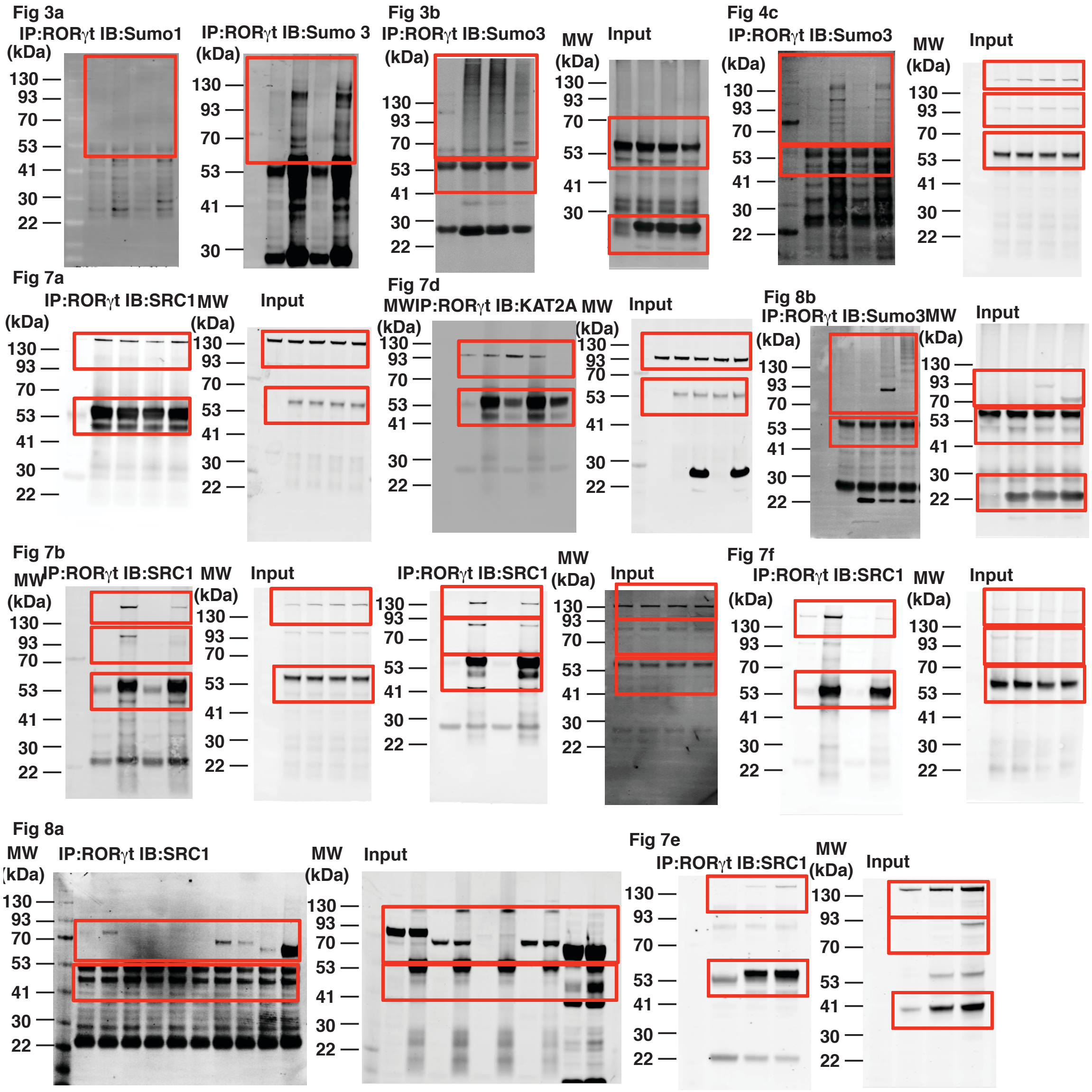

Supplementary Figure 9. Full-length images of immunoblot analyses.

Supplementary Table 1. List of primers used in this study.

Il17a loci	forward	ACATGAGTGCCGACAAACAACGGGT
	reverse	AGTCAGTGGGCTGCTGAATGACCCC
Il17f loci	forward	CACTTCATGCTGGTTTCTCCA
	reverse	GGAAGATCTGGTCCATTTTCC
Bcl2l1 loci	forward	TCAGAGCAAAAGCAAGAGCA
	reverse	GAGGAGGAGGGACGTTTTGT
beta hemoglobin loci	forward	GCTCTGGGTACTCCCTCTGA
	reverse	GCAAATGTGTTGCCAAAAAG
Il17a	forward	TTTAACTCCCTTGGCGCAAAA
	reverse	CTTTCCCTCCGCATTGACAC
Ifng	forward	ATGAACGCTACACACTGCATC
	reverse	CCATCCTTTTGCCAGTTCCTC
Csf2	forward	GGCCTTGGAAGCATGTAGAGG
	reverse	GGAGAACTCGTTAGAGACGACTT
Il17f	forward	TGCTACTGTTGATGTTGGGAC
	reverse	AATGCCCTGGTTTTGGTTGAA
Ccr6	forward	CCTGGGCAACATTATGGTGGT
	reverse	CAGAACGGTAGGGTGAGGACA
Ccl20	forward	GCCTCTCGTACATACAGACGC
	reverse	CCAGTTCTGCTTTGGATCAGC
Il22	forward	ATGAGTTTTTCCCTTATGGGGAC
	reverse	GCTGGAAGTTGGACACCTCAA
Ahr	forward	AGCCGGTGCAGAAAACAGTAA
	reverse	AGGCGGTCTAACTCTGTGTTC
Actb	forward	GAGTCCTACGACATCATCGCT
	reverse	CCGACATAGTTTGGGAAACAGT
