# Supplementary material for: Sumoylation of RORγt regulates TH17 differentiation and thymocyte development
Source: Nat Commun. 2018 Nov 19;9:4870. doi: 10.1038/s41467-018-07203-z (PMC6242824; doi:10.1038/s41467-018-07203-z)
Supplement: Supplementary file 2 — Peer Review file [file 41467_2018_7203_MOESM2_ESM.pdf]

Editorial Note: This manuscript has been previously reviewed at another journal that is not operating a transparent peer review scheme. This document only contains reviewer comments and rebuttal letters for versions considered at Nature Communications.

REVIEWERS' COMMENTS:

Reviewer #1 (Remarks to the Author):

The authors have addressed my major concerns.

Reviewer #2 (Remarks to the Author):

The authors have basically addressed the concerns in the revised manuscript with new data and revisions.
